# Unraveling the role of microRNA/isomiR network in multiple primary melanoma pathogenesis

**DOI:** 10.1038/s41419-021-03764-y

**Published:** 2021-05-12

**Authors:** Emi Dika, Elisabetta Broseghini, Elisa Porcellini, Martina Lambertini, Mattia Riefolo, Giorgio Durante, Phillipe Loher, Roberta Roncarati, Cristian Bassi, Cosimo Misciali, Massimo Negrini, Isidore Rigoutsos, Eric Londin, Annalisa Patrizi, Manuela Ferracin

**Affiliations:** 1 Department of Experimental, Diagnostic and Specialty Medicine (DIMES), University of Bologna, Bologna, Italy; 2 Dermatology Unit, IRCCS Azienda Ospedaliero-Universitaria di Bologna, Bologna, Italy; 3 Computational Medicine Center, Sidney Kimmel Medical College, Thomas Jefferson University, Philadelphia, PA 19107 USA; 4 Department of Translational Medicine and for Romagna, and “Laboratorio per le Tecnologie delle Terapie Avanzate” (LTTA), University of Ferrara, Ferrara, Italy; 5 CNR, Institute of Genetics and Biomedical Research, National Research Council of Italy, Milan, Italy

**Keywords:** Small RNAs, Melanoma

## Abstract

Malignant cutaneous melanoma (CM) is a potentially lethal form of skin cancer whose worldwide incidence has been constantly increasing over the past decades. During their lifetime, about 8% of CM patients will develop multiple primary melanomas (MPMs), usually at a young age and within 3 years from the first tumor/diagnosis. With the aim of improving our knowledge on MPM biology and pathogenesis, we explored the miRNome of 24 single and multiple primary melanomas, including multiple tumors from the same patient, using a small RNA-sequencing approach. From a supervised analysis, 22 miRNAs were differentially expressed in MPM compared to single CM, including key miRNAs involved in epithelial–mesenchymal transition. The first and second melanoma from the same patient presented a different miRNA profile. Ten miRNAs, including miR-25-3p, 149-5p, 92b-3p, 211-5p, 125a-5p, 125b-5p, 205-5p, 200b-3p, 21-5p, and 146a-5p, were further validated in 47 single and multiple melanoma samples. Pathway enrichment analysis of miRNA target genes revealed a more differentiated and less invasive status of MPMs compared to CMs. Bioinformatic analyses at the miRNA isoform (isomiR) level detected a panel of highly expressed isomiRs belonging to miRNA families implicated in human tumorigenesis, including miR-200, miR-30, and miR-10 family. Moreover, we identified hsa-miR-125a-5p|0|−2 isoform as tenfold over-represented in melanoma than the canonical form and differentially expressed in MPMs arising in the same patient. Target prediction analysis revealed that the miRNA shortening could change the pattern of target gene regulation, specifically in genes implicated in cell adhesion and neuronal differentiation. Overall, we provided a putative and comprehensive characterization of the miRNA/isomiR regulatory network of MPMs, highlighting mechanisms of tumor development and molecular features differentiating this subtype from single melanomas.

## Introduction

Melanoma is a malignant tumor that develops from transformed melanocytes. The incidence of cutaneous melanoma (CM) has been rising constantly in the past several decades, reaching an age-standardized rate of 17.8, 16.1, and 35.8 per 100,000 in Northern Europe, North America, and Australia-New Zealand, respectively (source IARC 2020).

CM accounts for 3–5% of all skin cancers, determining up to 65% of the skin cancer-related deaths^1^. The pathogenesis of CM is complex and poorly understood. Risk factors include environmental, genetic, and phenotypic factors such as ultraviolet (UV) exposure, fair phototypes, multiple dysplastic nevi, and a positive family/personal history of CM^2^. Among them, a family history of melanoma poses the highest risk for the development of this tumor^3,4^.

The occurrence of multiple primary melanomas (MPMs) in the same patient is thought to be related to a genetic susceptibility in association with lifestyle-correlated environmental factors. Thus, these patients represent a model of high-risk CM occurrence. The excision of a primary CM determines a risk up to 8.5%, to develop another CM in a lifetime. The frequency of MPMs is reported to range between 0.2% and 10%^5–7^ of all diagnosed melanoma cases.

The above-reported rates may be underestimated, due to the limited number of patients that undergoes a lifetime follow-up. Variability may also arise due to differences in environmental factors such as UV radiation exposure across geographical regions. The risk of a subsequent CM is highest in the first year following the diagnosis of the primary CM; however, this risk remains increased for at least 20 years^8^. The frequency of germline mutations in melanoma susceptibility genes (*CDKN2A* (cyclin-dependent kinase inhibitor 2A), *CDK4* (cyclin-dependent kinase 4), *MITF* (microphtalmia-associated transcription factor), *POT1* (protection of telomeres 1)/*ACD*/*TERF2IP*, *TERT*, and *BAP1*) is lower than expected in MPM patients^7,9–11^. The most frequent germline mutations in MPMs are in *CDKN2A* gene, occurring in 8–15% of subjects diagnosed with MPM without familial history and up to 40% of patients with hereditary CM (familial melanoma Orpha-618)^7,10,12,13^. Mutations in other susceptibility genes such as *CDK4*, *MITF*, and *POT1* are less frequently detected^14,15^.

Despite the increased risk of multiple tumor development, the debate about the prognosis of MPM patients is still opened. On one hand, Doubrovsky et al.^16^ observed a favorable prognosis in patients with MPM; on the other hand, a worse prognosis for these patients was recently reported by El Sharouni et al.^17^. Therefore, a better characterization of MPM pathogenesis and biological features is of the outmost importance.

The dysregulation of small noncoding RNAs, specifically microRNAs (miRNAs, 18–22 nucleotides in length), plays a significant role in tumorigenesis, including melanoma onset and progression^18^. MiRNAs regulate multiple and specific target genes, determining an oncogenic or tumor-suppressive function, being implicated in the proliferation, apoptosis, and tumor progression. Moreover, miRNA global expression profile faithfully classifies normal and pathological cells and tissues^19,20^, with the advantage to be obtainable also from formalin-fixed and paraffin-embedded (FFPE) tissues^21^.

Information on miRNA sequences are cataloged in miRBase database^22^, which reports a unique mature sequence for each miRNA, the so-called canonical form. Recently, evidences from deep-sequencing experiments suggested that miRNAs could have modifications in length and sequence in human tissues. These miRNA isoforms are called isomiRs^23^. The exact mechanisms by which isomiRs are generated is not fully understood; it is most likely due to alternative Drosha and/or Dicer cleavage of the precursor molecules^24^. Another mechanism that could explain isomiR biogenesis is posttranscriptional modifications made by nucleotidyl transferase, which adds nucleotides to pre-miRNA or mature miRNA ends^25–27^. IsomiRs can differentiate from the canonical counterpart in stability and abundance, and affects different downstream pathways^28^. It was suggested that canonical miRNAs and their isomiRs function cooperatively to target common biological pathways^23^.

In this study, we investigated the global miRNA and isomiR expression profile of MPMs using an unbiased small RNA-sequencing (RNA-seq) approach. A comparison of familial/non-familial MPM vs. single primary melanoma miRNome was established, to investigate the possible similarities. Moreover, MPM miRNA profile was assessed matching multiple tumors from the same patient. Finally, we characterized the miRNA/isomiR regulatory network in MPM by bioinformatic analysis.

## Materials and methods

### Clinical samples

A retrospective series of 47 samples from 29 patients was collected. Patients were selected among those referring to the melanoma center of the Dermatology Unit at Bologna University Hospital. The study was approved by Comitato Etico Indipendente di Area Vasta Emilia Centro - CE-AVEC, Emilia-Romagna Region (number 417/2018/Sper/AOUBo). Before study entry, all the patients provided written and voluntary informed consent for inclusion, collection, and use of clinical–pathological data and samples, and data privacy.

Histopathologic specimens were evaluated by two dermato-pathologists and were classified into three groups: benign nevi (BN), single primary CM, and MPM. Group 1 (*n* = 3) includes BN of three patients with no prior diagnosis of CM or non-melanoma skin cancer and a follow-up of at least 10 years. Group 2 (*n* = 35) includes MPM samples from 17 patients with prior diagnosis of ≥2 CMs. Three out of 17 patients had a positive family history of CM. Group 3 (*n* = 9) includes nine samples from CM patients with no history of prior CMs and a follow-up of at least 10 years.

Tumor and nevi samples were FFPE. For each sample, five to six tissue sections on glass slides were obtained. One section was stained with hematoxylin–eosin and was examined by an expert pathologist to select the tumor/nevus area, which was macrodissected before RNA extraction.

### RNA extraction

RNA was isolated from 10 µm-thick FFPE sections using miRNeasy FFPE kit (Qiagen, Hilden, Germany) according to the manufacturer’s instructions. Deparaffinization was performed with xylene followed by an ethanol wash. RNA was eluted in 30 µL of RNAse-free water and quantified, then stored at −80 °C.

### Small RNA sequencing

We analyzed 3 BN, 4 single CM, and 17 MPMs or familial melanomas from 8 different patients. The 24 small RNA libraries were generated using TruSeq Small RNA Library PrepKit v2 (Illumina, San Diego, CA, RS-200-0012/24/36/48), according to manufacturer’s indications. Briefly, 35 ng of purified RNA was ligated to RNA 3′- and 5′-adapters, converted into cDNA, and amplified using Illumina primers containing unique indexes for each sample. High Sensitivity DNA (HS-DNA) kit (Agilent Technologies, Santa Clara, CA, USA5067-4626) was adopted for library quantifications using Agilent Bioanalyzer (Agilent Technologies) and the 24 DNA libraries were combined in equal amount to generate a libraries pool.

Pooled libraries underwent to size selection employing magnetic beads (Agencourt, Beckman Coulter, Brea, CA) and amplicons with a length in the 130–160 bp range were recovered.

Finally, 20 pM of pooled libraries, quantified using the HS-DNA Kit (Agilent Technologies), were denatured, neutralized, and combined with a Phix control library (standard library normalizator). A 1.8 pM final concentration of pooled libraries (obtained by dilution with a dedicated buffer as described in Illumina protocol guidelines) was obtained and sequenced using NextSeq500/550 High Output Kit v2 (75 cycles) (Illumina, FC-404-2005) on the Illumina NextSeq500 platform.

Raw base-call data were demultiplexed using Illumina BaseSpace Sequence Hub and converted to FASTQ format. After a quality check with FastQC tool^29^, the adapter sequences were trimmed using Cutadapt^30^, which was also used to remove sequences shorter than 16 nucleotides and longer than 30 nucleotides. Reads were mapped using the STAR algorithm^31^. Only reads that mapped unambiguously to the reference genome retrieved from miRBase 21 database (at least 16 nucleotides aligned, with a 10% mismatch allowed) were used for the downstream analyses. Raw counts from mapped reads were obtained using the htseq-count script from the HTSeq tools^32^. Counts were normalized using DESeq2 bioconductor package^33^. Next-generation sequencing (NGS) raw data (FASTQ format) are available through European Nucleotide Archive (ENA) with the following accession number: PRJEB35819.

### Quantitative PCR

#### miRCURY LNA assay

RNA from 47 samples was converted to cDNA using miRCURY LNA RT kit (Qiagen, catalog number 339340). Reverse-transcription (RT) reaction was performed as follows: 2 µL miRCURY RT Reaction Buffer, 4.5 µL RNase-free water, 1 µL miRCURY RT Enzyme Mix, 0.5 µL UniSp6 spike-in, and 2 µL template RNA (5 ng/µL). RT cycling protocol consisted in 60 min at 42 °C, 5 min at 95 °C, and cooling at 4 °C. cDNA samples were stored at −20 °C. RT-quantitative PCR (qPCR) was performed using miRCURY LNA SYBER Green PCR kit (Qiagen, catalog number 339346) and primers from miRCURY LNA miRNA PCR Assays (Qiagen, catalog number 339306): hsa-miR-21-5p (catalog number YP00204230), hsa-miR-25-3p (catalog number YP00204361), hsa-miR-125b-5p (catalog number YP00205713), hsa-miR-146a-5p (catalog number YP00204688), hsa-miR-205-5p (catalog number YP00204487), hsa-miR-149-5p (catalog number YP00204321), hsa-miR-92b-3p (catalog number YP00204384), hsa-miR-200b-3p (catalog number YP00206071), hsa-miR-211-5p (catalog number YP00204009), hsa-miR-16-5p (catalog number YP00205702), and hsa-miR-125a-5p (catalog number YP00204339). For each miRNA target, cDNA was diluted at the ratio of 1 : 80 with the exception of miR-92b-3p, for which cDNA was diluted 1 : 4. Cycling program consisted in 10 min at 95 °C and 2-step cycling (40 cycles) of denaturation (10 s at 95 °C), and combined annealing/extension (60 s at 60 °C). Each assay was tested in triplicate. Raw Cq values were obtained from BioRad CFX software. Interplate calibrators were used to standardize miRNA Cq values across plates. miR-16-5p was selected as reference miRNA due to its stability across samples in NGS experiment. The calculation of relative expression was performed using 2^−ΔCt^ methods.

#### miSCRIPT assay (sum of 5′- and 3′-isoforms quantification)

RNA from 39 samples was reverse transcribed using miSCRIPT HiSpec Buffer from miSCRIPT II RT kit (Qiagen, catalog number 218161) according to the manufacturer’s instructions. Specifically, RT reaction was prepared in a total reaction volume of 10 µL with 2 µL miScript HiSpec Buffer, 1 µl miScript Nucleics Mix, 4 µL RNase-free water, 1 µL miScript Reverse Transcriptase Mix, and 2 µL template RNA (5 ng/µL). RT cycling protocol consisted in 60 min at 37 °C, 5 min at 95 °C, and cooling at 4 °C. cDNA samples were stored at −20 °C. RT-qPCR was performed using miSCRIPT SYBER Green PCR kit (Qiagen, cod. number 218073) and primers from miSCRIPT Primer Assays (Qiagen, catalog number 218300): hsa-miR-125a-5p (cod. number MS00003423), hsa-miR-200b-3p (cod. number MS00009016), RNA U6 (RNU6) (cod. number MS00033740). Reaction mix was prepared with 10 µL QuantiTect SYBR Green PCR Master Mix, 2 µL miScript Universal Primer, 2 µL miScript target Primer, 3 µL RNase-free water, and 3 µL cDNA template (1 : 40).

Cycling program consisted in 15 min at 95 °C and a 3-step cycling (40 cycles) of denaturation (15 s at 94 °C), annealing (30 s at 55 °C), and extension (30 s at 70 °C). Each assay was tested in triplicate. Raw Cq values were obtained from BioRad CFX software. Small nuclear RNU6 was used as reference gene. The calculation of relative expression was performed using 2^−ΔCt^ methods.

### IsomiR quantification

IsomiRs were identified in our NGS dataset of 24 samples as described in Loher et al.^34^. Briefly, sequence reads were quality trimmed using the Cutadapt tool^30^ and mapped unambiguously using SHRIMP2^35^ to the human genome assembly GRCh38. During the mapping, no insertions or deletions and, at most, one mismatch was permitted. IsomiRs were identified as done previously^34,36^. For The Cancer Genome Atlas (TCGA) isomiR analysis, short RNA-seq Aligned BAM files were downloaded from the Genomic Data Commons Data Portal (https://portal.gdc.cancer.gov/) for all 32 cancer types. IsomiR profiles were generated using the same approach as described in Loher et al.^34^. To simplify the labeling of the isomiRs, we used the annotation system developed by Loher et al.^34^. This nomenclature specifies the name of the canonical miRNA, the start site (5′-end) of the isomiR compared to the canonical miRNA sequence in miRBase, the end site (3′-end), and the eventual insertion of uracil. In particular, to annotate the start and end site of an isomiR, a negative (−) or positive sign (+) followed by the number of nucleotides is used to indicate the isomiR nucleotide shift in the 5’ or 3’ direction, respectively, if compared to the canonical miRNA sequence. Zero indicate the same terminus of the canonical miRNA sequence. We quantified isomiR abundances in reads per million. Only reads that passed quality trimming and filtering, and could be aligned exactly to miRNA arms were used in the denominator of this calculation. IsomiR targets were predicted using the RNA22 algorithm^37^ and targets were allowed to be present in the 5′-untranslated region (UTR), coding sequence, and 3′-UTR of the candidate mRNA. We selected only those targets that had a *p*-value < 0.01 and a predicted binding energy < −16 kcal/mol, while also allowing G : U wobbles and bulge’s within the seed region.

### Statistical analysis

Normalized sequencing data were imported and analyzed in Genespring GX software (Agilent Technologies). Differentially expressed miRNAs were identified using a fold-change >1.5 filter and moderated *t*-test (false discovery rate 5% with Benjamini–Hochberg correction) in CM vs. MPM comparison, and using fold-change >1.2 and paired *t*-test (*p* < 0.05) in first vs. second MPM comparison. Cluster analysis was performed using Manhattan correlation as a similarity measure. Principal component analysis (PCA) was performed on 24 samples using all human miRNAs detected by NGS analysis (*n* = 1629).

Graphpad Prism 6 (GraphPad Software, San Diego, CA) was used for statistical analyses. Group comparison was performed using unpaired *t*-test, when data had a normal distribution, with or without Welch’s correction according to the significance of the variance test. Data that did not present a normal distribution were compared using Mann–Whitney non-parametric test. Paired *t*-test was used to compare miRNA expression level in the first and second melanomas from the same patient. Two-tailed *p*-values are used in text and figures. Association of gene expression with overall survival in TCGA skin cutaneous melanoma (SKCM) cohort was obtained using the Oncolnc website (http://www.oncolnc.org) and log rank test was used to calculate the *p*-value.

### Pathway analysis

Pathway and network analysis of differentially expressed miRNAs, miR-125a-5p isoforms, and their targets was investigated using the web-based software MetaCore (Clarivate, Philadelphia, PA). A *p*-value of 0.05 was used as a cutoff to determine significant enrichment.

## Results

### Patient characteristics

Demographic, clinical, and pathological features of 47 samples from 29 individuals are summarized in Table 1. Samples from 15 individuals (8 with MPMs, 4 with CMs, and 3 with BNs) were investigated with NGS (*N* = 24). All the samples were used for the validation of NGS results. Nine patients had single CM and 10 years of follow-up; 17 developed more than one primary melanoma in an average time of 33 months (range 3–98). MPM patients were tested for germline genetic alterations in *CDKN2A*, *CDK4*, and *MITF* gene^38^, and only one patient was found to have a germline *CDKN2A* mutation (c.249 C > A p.His83Gln exon 2) of unknown clinical significance.

**Table 1:** Patient characteristics.

	Benign nevi (BN)	Cutaneous melanoma (CM)	Multiple primary melanoma (MPM)		
			MPM 1st	MPM 2nd	MPM 3rd
Gender (*n*, %)					
Male	0 (0%)	3 (33.3%)	13 (76.5%)		
Female	3 (100%)	6 (66.7%)	4 (23.5%)		
Total	3	9	17		
Histology (*n*, %)					
Compound melanocytic nevus	2 (66.7%)	-	-	-	-
Dermal nevus	1 (33.3%)	-	-	-	-
Superficial spreading melanoma	-	4 (44.5%)	15 (88.2%)	16 (94.1%)	1 (100%)
Superficial spreading melanoma with vertical growth phase	-	3 (33.3%)	1 (5.9%)	-	-
Nodular melanoma	-	2 (22.2%)	1 (5.9%)	-	-
Nevus-associated melanoma	-	0	-	1 (5.9%)	-
Age at first diagnosis (*n*, %)					
<50	-	7 (77.8%)	8 (47%)		
≥50	-	2 (22.2%)	9 (53%)		
Mean (range)	-	59 (29–85)	53 (30–80)		
Localization (*n*, %)					
Trunk	3 (100%)	6 (66.7%)	13 (76.5%)	14 (82.4%)	1 (100%)
Limbs	-	2 (22.2%)	3 (17.6%)	3 (17.6%)	-
Head and neck	-	1 (11.1%)	1 (5.9%)	-	-
Breslow thickness (*n*, %)					
<0.8 mm	-	1 (11.1%)	12 (70.6%)	17 (100%)	1 (100%)
≥0.8 mm	-	8 (88.9%)	5 (29.4%)	0 (0%)	-
Family history of melanoma (*n*, %)					
Yes	-	-	3 (17.6%)		
No	-	-	14 (82.4%)		

### The miRNA profile of MPM

**Fig. 1:**
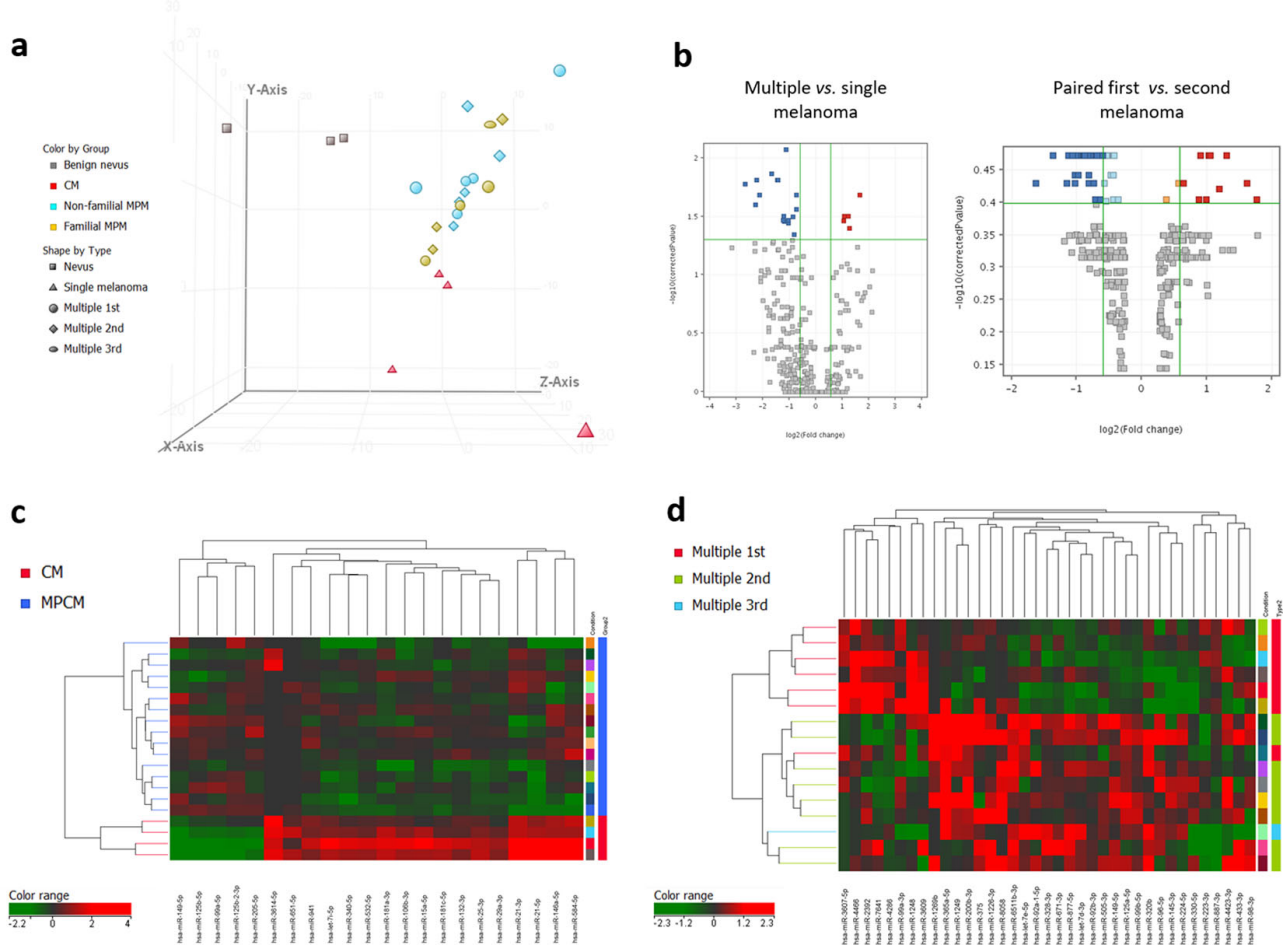
Single and multiple melanoma classification based on the miRNA profile. **a** Unsupervised principal component analysis (PCA) of 24 samples based on the expression profile of all the miRNAs detected at NGS analysis. Familial (yellow) and non-familial (cyan) multiple primary melanomas display a similar microRNA profile, which is different from single cutaneous melanoma (red) and benign nevi (gray). **b** Cluster analysis and heatmap representation of multiple and single melanoma based on the expression of 22 differentially expressed miRNAs (moderated *t*-test, adjusted *p* < 0.05). Red and green color represent the increased or reduced expression across samples. **c** Cluster analysis and heatmap representation of the first and second melanomas from the same patient based on the expression of 37 differentially expressed miRNAs (paired *t*-test, *p* < 0.05). Red and green color represent the increased or reduced expression across samples. **d** Volcano plot showing the differentially expressed miRNAs at the selected *p*-value and fold-change combinations.

The global miRNA profile of 17 MPMs, obtained from 8 patients, was analyzed using a small RNA-seq approach. For each MPM patient, we analyzed the first and second primary tumor, and for one case also a third one. Three patients had a family history of melanoma. We compared the global miRNA profile of 17 MPMs vs. 4 single melanomas and 3 BN. From the small RNA-seq data, we identified 1629 mature miRNAs expressed in melanoma and nevus cells. The unsupervised PCA of all miRNAs and all samples (*n* = 24) revealed that familial and non-familial MPMs have a greatly overlapping miRNA profile (Fig. 1a), which is different from single CM and BN. Indeed, a statistical comparison between familial and non-familial MPMs did not provide any significant result. Therefore, we considered familial and non-familial melanomas as a unique group in all subsequent analyses. From the PCA analysis, we can already observe that MPMs displayed an miRNA profile intermediate between BN and CMs. When we compared multiple and single melanoma tumors, we obtained a markedly different miRNA expression profile and a list of 22 miRNAs differentially expressed (adjusted *p* < 0.05, Supplementary Table 1), which are represented with a Volcano plot in Fig. 1b. Cluster analysis of these samples based on the expression of the 22 differentially expressed miRNAs confirmed the separation between single and multiple tumors (Fig. 1c). We evaluated the miRNA profile in MPM groups using a paired statistical analysis. Despite the similarities between the two matching MPMs, a variation in miRNA expression was observed (Fig. 1b). Specifically, 37 miRNAs were differentially expressed between the first and second MPM (paired *t*-test, *p* < 0.05, Supplementary Table 2), whose expression clearly separate the groups in the cluster analysis (Fig. 1d).

### Validation of miRNA differential expression in single and multiple primary melanomas, and paired primary tumors from the same patient

Nine miRNAs were selected for an independent validation using quantitative RT-PCR in 47 samples including BN, CM, and first and second MPM. Specifically, we included six miRNAs differentially expressed between CM and MPM (miR-21-5p, miR-25-3p, miR-125b-5p, miR-146a-5p, miR-205-5p, and miR-149-5p) and four that are dysregulated between the first (MPM 1st) and second (MPM 2nd) melanoma from the same MPM patient (miR-149-5p, miR-92b-3p, miR-200b-3p, and miR-125a-5p).

According to the small RNA-seq results, an upregulated expression of miR-21-5p, miR-25-5p, and miR-146a-5p, and a downregulated expression of miR-125b-5p, miR-149-5p, and miR-205-5p in CM compared to MPM were expected. In MPM samples, all selected miRNAs are upregulated in the MPM 2nd compared to MPM 1st. In the validation experiment, we included also miR-211-5p, whose genetic locus is located inside the *melastatin-1*/*TRPM1* (transient receptor potential cation channel, subfamily M, member 1 protein) gene and whose expression is particularly high in nevi. The expression of this miRNA was higher in BN, with borderline statistical significance when compared to CM or MPM in our NGS data.

**Fig. 2:**
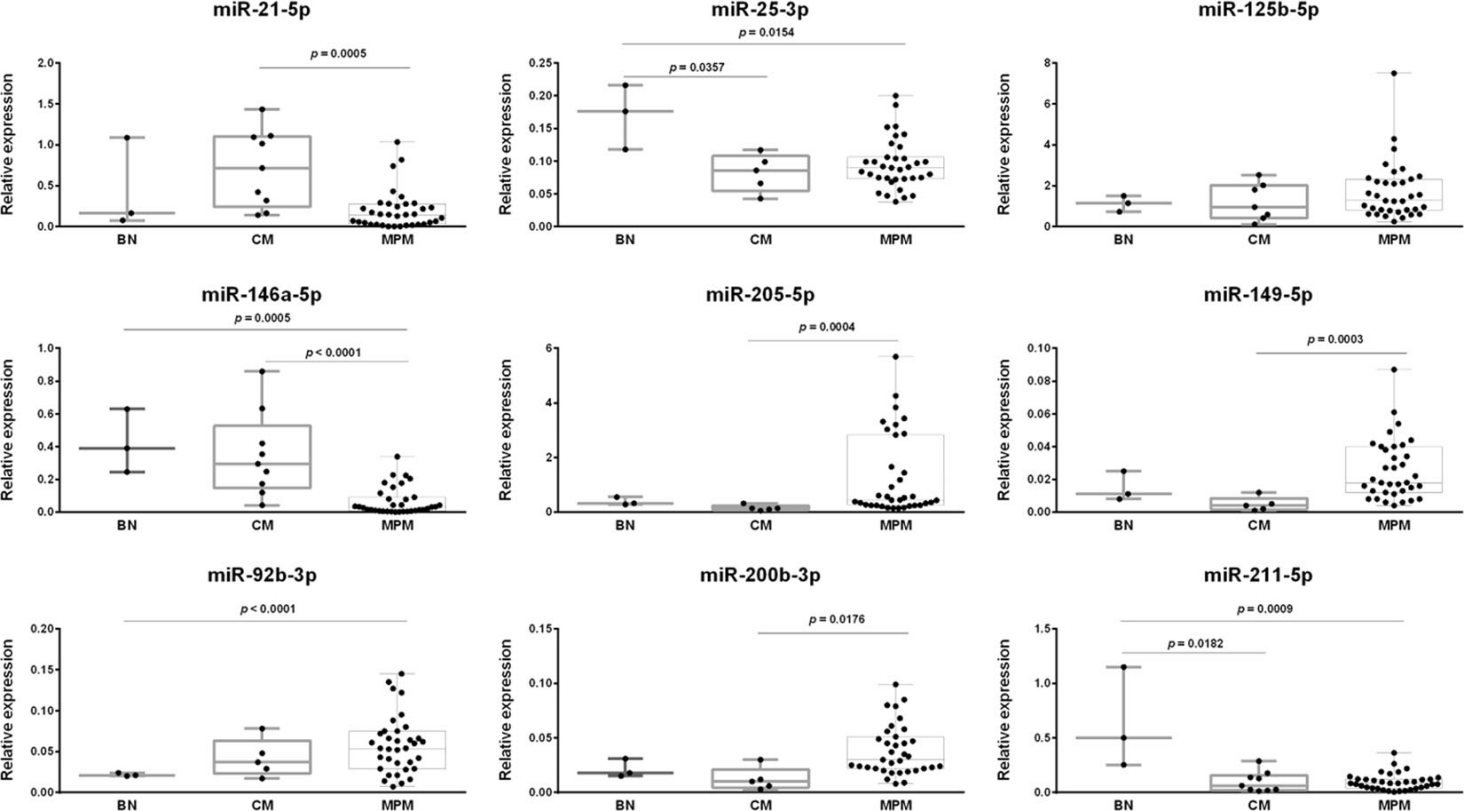
Differential microRNA expression in benign nevi (BN), cutaneous melanoma (CM), and in multiple primary melanoma (MPM). Box and whiskers graph representation of nine microRNAs differentially expressed in single and multiple primary melanomas (*P* < 0.05). MPM shows higher expression levels of miR-205-5p, miR-200b-3p, and miR-149-5p compared to CM, and higher expression of miR-92b-3p compared to BN. MPM downregulates miR-21-5p compared to CM and miR-146a-5p compared to CM and BN. BN upregulates miR-25-3p and miR-211-5p compared to CM and MPM. Each miRNA was tested in triplicate by quantitative RT-PCR. Relative miRNA expression was normalized on invariant miR-16-5p. The bar shows minimum and maximum values; superimposed points represent all individual values.

The validation was performed in a cohort of 29 patients, as described in Table 1, using miR-16-5p as a reference gene due to its invariant expression in NGS data. Expression distributions of selected miRNAs in BN, CM, and MPM samples are represented in Fig. 2. The significant upregulation of miR-21-5p in CM vs. MPM was confirmed. A statistically significant miR-25-3p downregulation in CM and MPM compared to BN was observed, while miR-146a-5p was downregulated in MPM compared to BN and CM. A similar expression level in CM and BN, and a trend toward increased expression in MPM were obtained for miR-125b-5p. A significant upregulation of miR-200b-3p, miR-205-5p, and miR-149-5p was observed in MPM compared to CM. As expected, miR-211-5p is progressively downregulated in multiple and single melanomas (Fig. 2).

**Fig. 3:**
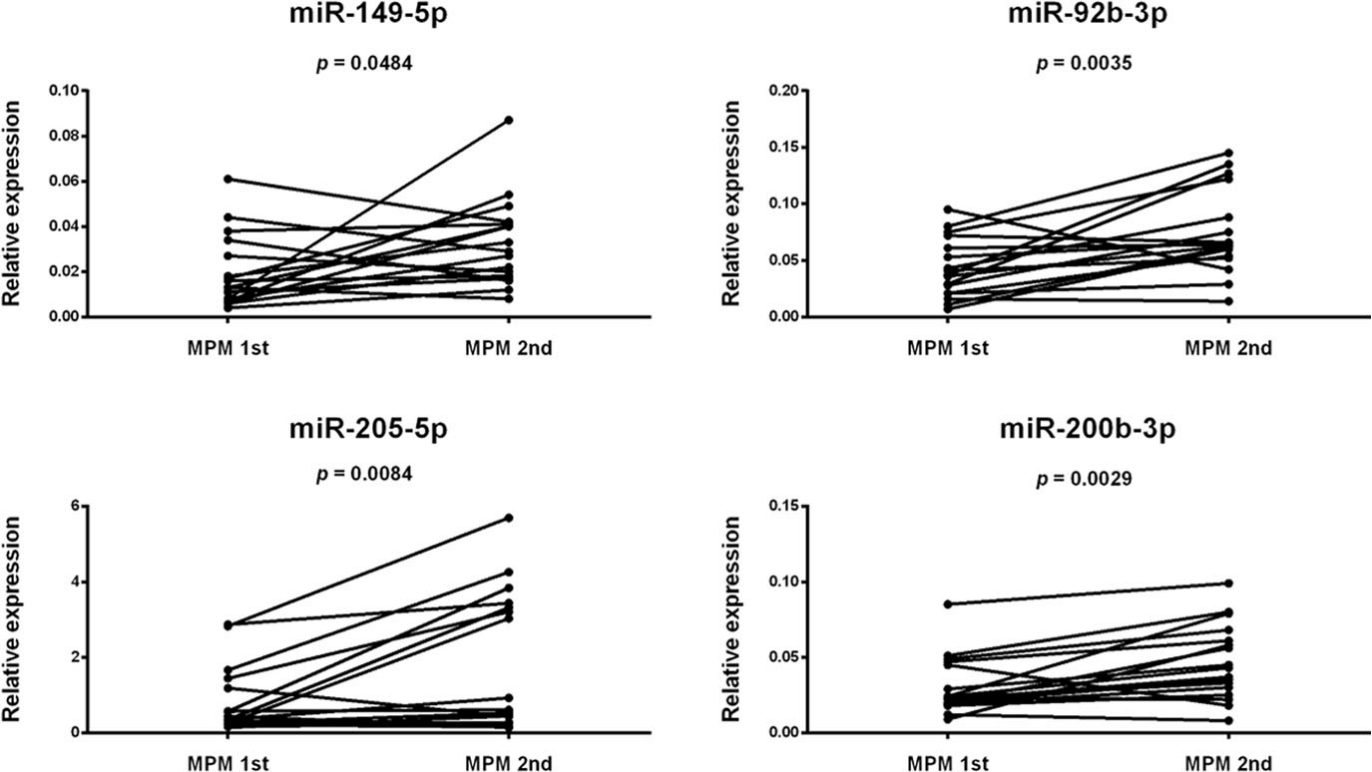
Differential microRNA expression in first vs. second melanoma from the same patient. Before–after plot showing the paired expression of 4 selected microRNAs in 17 multiple primary melanoma (MPM) patients. miR-92b-3p, miR-205-5p, miR-200b-5p, and miR-149-5p are significantly downregulated in the first melanoma compared to the second melanoma. Each miRNA was tested in triplicate by quantitative RT-PCR. Relative miRNA expression was normalized on invariant miR-16-5p. Paired t-test *p*-value is reported.

The differential expression of miR-149-5p, miR-92b-3p, miR-200b-3p, and miR-205-5p between paired first and second melanomas from the same patient is represented in Fig. 3. The expected significant upregulation for miR-149-5p, miR-92b-3p, miR-205-5p, and miR-200b-3p in MPM 2nd tumor was confirmed in this larger group of samples.

### Functional annotation of MPM miRNA signature

The list of 22 miRNAs differentially expressed in multiple vs. single melanomas was uploaded in MetaCore software to identify both the pathways that are significantly regulated by these miRNAs (Supplementary Table 3) and the most significant miRNAs/targets networks (Supplementary Fig. 1a).

**Fig. 4:**
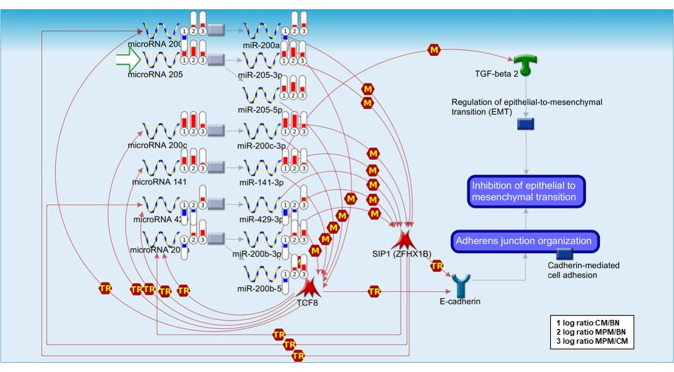
MetaCore pathway analysis showing the involvement of differently expressed miRNAs in epithelial–mesenchymal transition (EMT). EMT pathway representation with regulating miRNAs. Log ratio of miRNA expression level in CM/BN (1), MPM/BN (2), and MPM/CM (3) is visualized on the maps as a thermometer-like figure. Upward thermometers have a red color and indicate upregulated signals, and downward (blue) ones indicate downregulated expression level of specific microRNAs. “M” indicates microRNA binding (regulation of gene expression by binding of microRNA to target mRNA), whereas “TR” indicates Transcription regulation (physical binding of a transcription factor to target gene’s promoter). MPM showed higher expression levels of microRNAs involved in the inhibition of epithelial–mesenchymal transition (EMT), including miR-205-5p and miR-200b-3p. (BN, benign nevi; CM, cutaneous melanoma; MPM multiple primary melanoma).

MPMs were found to have a higher expression of epithelial–mesenchymal transition (EMT)-associated miRNAs (miR-149-5p, miR-200 family, and miR-205-5p) compared to single CM and even nevi (Figs. 2 and 4). miR-149-5p inhibits FOXM1^39^, whereas miR-200 family and miR-205-5p target *ZEB1/TCF8* and *ZEB2/SIP1* genes, and by doing so they inhibit the EMT pathway. Therefore, this pathway appears to be specifically activated in single melanomas (Fig. 4).

From MetaCore network analysis, three hub genes (*TLR4*, *ITGA6*, and *BTG2*) were identified as targeted by multiple miRNAs, either up- or downregulated in multiple melanomas. When we assessed the association of *TLR4*, *ITGA6*, and *BTG2* gene expression with melanoma prognosis, we observed that their higher expression (median cutoff) was significantly associated with a worse overall survival in TCGA SKCM cohort of 458 samples (Supplementary Fig. 1b).

### IsomiR analysis revealed that a miR-125a-5p isoform is dysregulated in MPM

Interestingly, miR-125a-5p differential expression in MPM was not confirmed by qPCR technology and we wondered about a possible explanation. We observed that the reads generated by the small RNA-seq experiment and attributed to mature miR-125a-5p following the standard matching pipeline were actually shorter by 1, or most frequently 2 nucleotides (lack of GA at the 3′-end) in all samples (Supplementary Fig. 2a).

We analyzed the miRNA variants (isomiRs) expression level in our NGS data. We found 90 miRNAs with sequence and length heterogeneity, generating 324 different isomiRs, and 40 canonical miRNAs without any isomiR. In addition, we found and excluded 40 isomiRs named “orphan,” because their canonical miRNA sequences could not be detected in our NGS data. Orphan isomiRs can originate by the addition of non-templated base pairs, from either uridylation, adenylation, or other modifications. As these bases are posttranscriptionally added, the full “orphan” isomiR cannot be found in the genome. In addition, Cloonan et al.^23^ suggested that orphan isomiRs could also be mis-annotated mature miRNAs. Given their still unknown biological role, we did not carry out any further investigation on this class.

For each isomiR, we calculated the average expression in all sequenced samples and the ratio between each isomiR and its canonical miRNA. As the low expressed isomiRs are bound to have a marginal role, we focused on highly expressed isomiRs that could potentially affect the target gene regulation of the canonical counterpart. We obtained a panel of 17 isoforms that are 3- to 10-fold more abundant than their canonical forms (Table 2). Among them, we noticed an enrichment of isomiRs belonging to three important miRNA family: miR-30 family, miR-200 family, and miR-10 family. For each family member, we analyzed the expression of its isomiRs and canonical forms (Supplementary Figs. 3–5). The most representative miR-30 family isomiRs are 3′-variants with the addiction of 1 or 2 nucleotides (Supplementary Fig. 3). On the contrary, the most expressed miR-10 family isomiRs are shorter 3′-isoforms (−1 or −2 nucleotides) (Supplementary Fig. 4). miR-200 family presented highly expressed 3′-isoforms, both with addiction or deletion of nucleotides (Supplementary Fig. 5).

**Table 2:** IsomiRs most represented in melanoma and nevi small RNA-sequencing data.

IsomiR	Type	IsomiR expression (mean)	Canonical miRNA expression (mean)	Ratio isomiR/canonical miRNA
hsa-miR-141-3p|0|−1	End-site isomiR	200.33	8.65	23.15
hsa-miR-222-3p|0|+3	End-site isomiR	154.64	9.05	17.08
hsa-miR-30a-5p|0|+2	End-site isomiR	211.95	13.33	15.90
hsa-miR-125a-5p|0|−2	End-site isomiR	765.69	56.46	13.56
hsa-miR-30d-5p|0|+2	End-site isomiR	416.87	41.32	10.09
hsa-miR-10b-5p|0|−1	End-site isomiR	2950.88	357.98	8.24
hsa-miR-27a-3p|0|−1	End-site isomiR	167.78	30.55	5.49
hsa-miR-30a-5p|0|−2	End-site isomiR	72.12	13.33	5.41
hsa-miR-222-3p|0|+4	End-site isomiR	46.98	9.05	5.19
hsa-miR-19b-3p|0|−1	End-site isomiR	52.06	11.59	4.49
hsa-miR-30c-5p|0|+1	End-site isomiR	151.53	34.28	4.42
hsa-miR-26b-5p|0|+1	End-site isomiR	172.47	39.32	4.39
hsa-miR-222-3p|0|+2	End-site isomiR	36.83	9.05	4.07
hsa-miR-10a-5p|0|−1	End-site isomiR	636.58	184.52	3.45
hsa-miR-30a-5p|0|+1	End-site isomiR	45.68	13.33	3.43
hsa-miR-30d-5p|0|+1	End-site isomiR	140.07	41.32	3.39
hsa-miR-200b-3p|0|+1	End-site isomiR	44.86	13.26	3.38

In the group of 17 miRNAs with highly expressed isomiRs, we found two isomiRs whose canonical miRNA was differentially expressed in first vs. second MPMs: miR-125a-5p and miR-200b-3p.

**Fig. 5:**
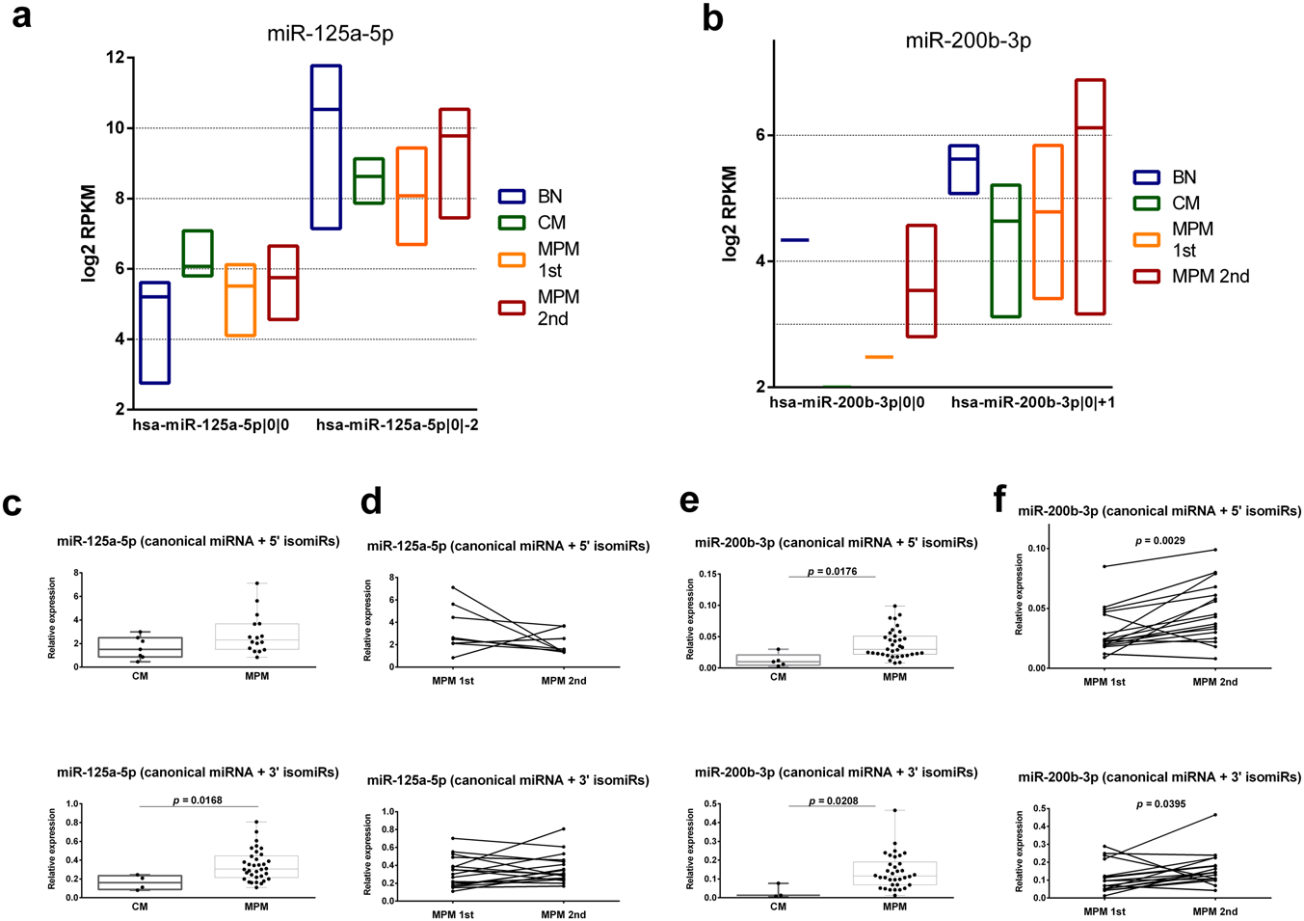
Comparison of miR-125a-5p and miR-200b-3p isomiR expression in benign nevi (BN), cutaneous melanoma (CM), and multiple primary melanoma (MPM). **a** Floating bar chart of miR-125a-5p isomiR expression. Canonical miR-125a-5p (hsa-miR-125a-5p|0|0) shows a lower expression level and opposite expression trend in BN, CM, and MPM if compared to its shorter isomiR (hsa-miR-125a-5p|0|−2). Bars represent min–max and median values. **b** Floating bar chart of miR-200b-3p isomiR expression showing the same expression trend for canonical miR-200b-3p (hsa-miR-200b-3p|0|0) and its longer isomiR (hsa-miR-200b-3p|0|+1) in MPM groups. Canonical miR-200b-3p was not detected in CM samples. Bars represent min–max and median values. **c** Box and whiskers graph representation of canonical miR-125a-5p expression assessed with miRCURY LNA assay and overall expression of all miR-125a-5p isoforms, detected using miSCRIPT assay. Results show that the combined expression of miR-125a-5p isoforms of levels is higher in MPM compared to CM. The bar shows minimum and maximum values; superimposed points represent all individual values. **d** Before–after plot of canonical miR-125a-5p expression (miRCURY LNA assay) and all miR-125a-5p isoforms (miSCRIPT assay) show an opposite trend in the first and second melanoma from the same patient. **e** Box and whiskers graph representation of canonical miR-200b-3p expression assessed with miRCURY LNA assay and overall expression of all miR-200b-3p isoforms, detected using miSCRIPT assay. Results show that in both there is a higher expression in MPM compared to CM. The bar shows minimum and maximum values; superimposed points represent all individual values. **f** Before–after plot of canonical miR-200b-3p expression (miRCURY LNA assay) and all miR-200b-3p isoforms (miSCRIPT assay) show both a higher expression in the second melanoma compared to the first and from the same patient.

Quite peculiarly, miR-125a-5p canonical form and hsa-miR-125a-5p|0|−2 isoform show opposite expression trends in nevi, single primary melanomas, and MPMs (Fig. 5a). On the other hand, hsa-miR-200b-3p|0|+1 and its canonical form showed the same upregulated expression in MPM 2nd compared to the MPM 1st (Fig. 5b).

**Fig. 6:**
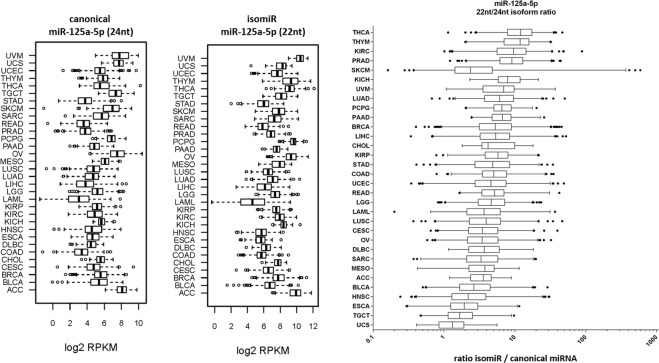
Canonical hsa-miR-125a-5p|0|0) and hsa-miR-125a-5p|0|−2 isomiR expression across 32 TCGA tumor types. miR-125a isomiR is most expressed in many cancer types. Box and whiskers graphs of canonical and shorter isoform of miR-125a-5p show variable expression levels, represented here as log2 RPKM data, across 32 different cancer type. miR-125a-5p isoform is most abundant in many cancer types and shows a specifically high canonical/isoform ratio in the melanoma (SKCM) group. The bar shows 1–99 percentile values. TCGA abbreviations: ACC, adrenocortical carcinoma; BLCA, bladder urothelial carcinoma; BRCA, breast invasive carcinoma; CESC, cervical squamous cell carcinoma and endocervical adenocarcinoma; CHOL, cholangiocarcinoma; COAD, colon adenocarcinoma; DLBC, lymphoid neoplasm diffuse large B-cell lymphom; ESCA, esophageal carcinoma; HNSC, head and neck squamous cell carcinoma; KICH, kidney chromophobe; KIRC, kidney renal clear cell carcinoma; KIRP, kidney renal papillary cell carcinoma; LAML, acute myeloid leukemia; LGG, brain lower grade glioma; LIHC, liver hepatocellular carcinoma; LUAD, lung adenocarcinoma; LUSC, lung squamous cell carcinoma; MESO, mesothelioma; OV, ovarian serous cystadenocarcinoma; PAAD, pancreatic adenocarcinoma; PCPG, pheochromocytoma and paraganglioma; PRAD, prostate adenocarcinoma; READ, rectum adenocarcinoma; SARC, sarcoma; SKCM, skin cutaneous melanoma; STAD, stomach adenocarcinoma; TGCT, testicular germ cell tumors; THCA, thyroid carcinoma; THYM, thymoma; UCEC, uterine corpus endometrial carcinoma; UCS, uterine carcinosarcoma; UVM, uveal melanoma.

We studied two different technical approaches for isomiR quantification based on commercial RT-qPCR kits (miRCURY LNA and miSCRIPT), in all samples. Specifically, miRCURY LNA assay design was used to quantify the canonical form (including the 5′-isoforms, which are not abundant/detected in our NGS data), whereas miSCRIPT assay was used to detect all the isoforms, as a consequence of the use of a universal 3′-primer during miRNA amplification (Supplementary Fig. 2b). As expected, the two assays validated miR-200b-3p differential expression, providing the same results obtained by NGS data. In fact, both the canonical miRNA and hsa-miR-200b-3p|0|+1 showed an upregulation in MPM 2nd compared to the MPM 1st (Fig. 5e, f). For miR-125a-5p, miRCURY LNA assay did not quantify the predominant 3′-isomiRs; therefore, the NGS data could not be validated (Fig. 5c, d). Results revealed a lack of variation between single and multiple melanomas, and a higher expression in the first vs. second melanoma using (Fig. 5c, d). Given the high predominance of the of hsa-miR-125a-5p|0|−2 isoform in our samples, we assumed that the miSCRIPT assay could provide a bona fide quantification of hsa-miR-125a-5p|0|−2 isomiR. As expected, an increase in miR-125a-5p level in MPMs vs. CMs and in the second tumor from the same patient was observed (Fig. 5c, d). As a further confirmation of our findings, we examined the expression of hsa-miR-125a-5p|0|−2 (isomiR, 22 nt long) and 0|0 (canonical miRNA, 24 nt long) isoforms across all TCGA tumor types and discovered an overall higher expression of the shorter form in human cancers and a specifically altered ratio of the two forms in SKCM (CM cohort), which shows the largest variation (Fig. 6).

### miR-125a-5p miRNA/isomiR regulated different target genes

We ran a bioinformatics analysis to predict the impact of miR-125a-5p 3′-isomiR in target gene binding. We used RNA22 algorithm to obtain the list of putative pairing sites for miR-125a-5p canonical form (*N* = 1342) and its 3′-isoform miR-125a-5p|0|−2 (*N* = 971) (Supplementary Table 4). The shorter miR-125a-5p isoform loses the ability to bind a fraction of canonical miR-125a-5p|0|0 targets (Supplementary Table 5). The predicted target genes were submitted for functional annotation to MetaCore suite. Significantly enriched pathways and networks were identified for common and specific targets of canonical miR-125a-5p canonical (miR-125a-5p|0|0) and its 3′-isomiR (miR-125a-5p|0|−2) (Supplementary Table 6), revealing a loss of targeting for miR-125a-5p|0|−2 of genes involved in nervous system development, neurogenesis, and neuronal differentiation. IsomiR miR-125a-5p|0|−2 no longer targets key genes involved in cell adhesion and migration (Ephrin receptors, *Netrin 1*) or intracellular signaling (*PIK3C2B*).

## Discussion

The risk of melanoma development is influenced by environmental and genetic factors^2^. Families with history of melanoma could have a germline mutation that confers hereditary susceptibility and this is particularly demonstrated in families where more than one member develop MPMs^40,41^. Nowadays, most studies report a very low prevalence of mutation in *CDKN2A* and *CDK4* genes in multiple melanoma patients, especially in Southern Europe countries^9^. Although MPM patients often report similar sun exposure experiences, the high percentage of atypical nevi in these patients and their family members, the frequent family history of melanoma, as well as the early onset of melanoma (young age) suggest that predisposing factors are involved in the development of the disease^42^. However, cases of MPM in individuals without familial history of melanoma have also been reported. In these cases, germline mutations in melanoma predisposing genes are rarely detected^7,9–11^. Therefore, it is evident that some other genetic or epigenetic factors are active in MPM to fuel multiple events of melanocytic transformation.

In this study, we provide the first comprehensive molecular characterization of MPMs by analyzing their miRNome with a small RNA-seq approach. The global miRNA expression reflects the mRNA expression of cells and tissues, with the advantage of being assessable in FFPE tissues, which is the typical available tissue for thin melanomas. Our analysis revealed a specific expression pattern of multiple melanoma tumors when compared to single CM. MPM miRNome is more similar to BN, thus suggesting a less aggressive and more differentiated phenotype. We observed no distinct miRNA pattern in MPMs with or without a recognized family history of melanoma. We validated a panel of miRNAs in a larger cohort, including also multiple tumors from the same patient, obtaining a panel of miRNAs differentially expressed in tumors from the same patient.

Many studies have attempted to define the prognosis of patients with MPMs and results are still controversial, with studies stating that developing multiple melanomas is associated with worse prognosis or the opposite^43–45^. Recently, Grossman et al.^46^ revealed the potential reasons for these controversies by analyzing with proper multivariate statistical analysis, the Surveillance, Epidemiology, and End Results data, using a single matching method and demonstrating that there is no substantial difference among single and multiple melanoma patients. In this study, we provide the evidence that MPMs, from a biological point of view, have a less invasive phenotype as pointed out by the main regulatory pathways activated in these tumors, thus providing further elements of discussion to support MPM’s less aggressive behavior. It is worth mentioning that miRNAs known to inhibit EMT (e.g., miR-200 family, miR-205, miR-149)^39,47,48^ are more expressed in MPMs compared to that in single melanomas. Tumor cells promote EMT to escape from their microenvironment and migrate to new locations, and give rise to metastases^49^. The acquisition of a mesenchymal phenotype promotes the production of extracellular matrix proteins, the resistance to apoptosis, the invasiveness, and the migration^50^. EMT results from the loss of cell-to cell junctions, induced by the loss of E-cadherin; the process is mediated by transcription factors, including SNAIL, SLUG, SIP1, and E2A, and affected by regulatory proteins such as TGFβ, EGF, PDGF, ERK/MAPK, PI3K/AKT, SMADS, RHOB, β‐catenin, LEF, RAS, C‐FOS, integrins β4, and integrin α5^51^. Despite their origin from neural crest-derived melanocytes, EMT has been reported in melanoma cells, where it promotes the metastatic phenotype of malignant melanocytes^52,53^. Caramel et al.^53^ described a switch in the expression of embryonic EMT inducers that drives the development of malignant melanoma. They found that EMT-inducing transcription factors undergo a profound reorganization in favor of TWIST1 and ZEB1 during melanoma metastatization^53^. Another work has demonstrated that ZEB2 is important for migration and melanocyte differentiation in a mice model^54^. The inhibition of FOXM1 leads to an antitumor effect in melanoma^55^. Moreover, many studies described the loss of E-cadherin in melanoma^56,57^; E-cadherin is normally expressed by melanocytes and mediates the adhesion between melanocytes and keratinocytes^58^. Hao et al.^59^ observed a switch from E-cadherin to N-cadherin expression in melanoma progression, a process regulated by phosphatidyl inositol 3-kinase (PI3K) and phosphatase and tensin homolog (PTEN) through TWIST and SNAIL. In addition, the ectopic expression of Cadherin 1 was associated with the downregulation of adhesion receptors, such as MCAM/MUC18 and β3 integrin subunit, resulting in suppression of melanoma cells invasion^60^.

Consistently, we examined the main cellular hubs regulated by MPM-specific miRNAs and discovered that they are centered in Toll-like receptor 4 (TLR4), integrin α6 (ITGA6), and BTG2 proteins. miRNAs regulating these hubs are mostly downregulated in MPMs and high expression of these three genes is associated with a more favorable prognosis in TCGA SKCM cohort.

ITGA6, also known as CD49f, is a transmembrane glycoprotein adhesion receptor that mediates cell–matrix and cell–cell interactions. ITGA6 was identified and described as an important stem cell biomarker. Indeed, it is the only common gene expressed in embryonic stem cells, neural stem cells, and hematopoietic stem cells^61,62^. It is also expressed in more than 30 stem cell populations, including cancer stem cells^63^. ITGA6 can combine with other integrins such as integrin β1 and integrin β4 to form respectively integrin VLA-6 and TSP180. The role of ITGA6 in melanoma is not clear but our observation point toward its upregulation in MPMs upon miR-25 and miR-29 downregulation.

BTG2 is part of the anti-proliferative BTG/TOB family and its expression is p53 dependent^64^. This protein is involved in several cellular processes, including cell cycle regulation, DNA damage repair, cell differentiation, proliferation, and apoptosis. However, its role is often cell-type dependent^65^. In fact, BTG2 inhibits proliferation and migration, acting as a tumor suppressor protein, in gastric cancer cells^66^ and in lung cancer cells^67^, whereas in bladder cancer it promotes cancer cell migration^68^. In B16 melanoma cells, it was shown that miR-21 promoted a metastatic behavior through the downregulation of many tumor suppressor proteins, including PTEN, PDCD4, and BTG2^69^. In MPMs, we observe the downregulation of several miRNAs targeting BTG2, including miR-21-5p, miR-146a-5p, miR-132-3p, and miR-15a-5p. Therefore, an upregulation of *BTG2* expression is to be expected.

TLR4 belongs to the TLR family and plays an important role in inflammation and cancer. TLR4 protein is expressed at very low levels in melanoma cells in vivo (Human protein atlas) but its activation has been reported to promote an inflammatory microenvironment and tumor progression in vitro^70^. In addition, TLR4 is associated with induction of proliferation and migration of melanoma cells^71^. TLR4 plays an important role in melanoma as well, because it interacts with TRIM44, a negative prognostic factor in melanoma. In particular, TRIM44 binds and stabilizes TLR4, leading to the activation of AKT/mTOR signaling, which results in EMT promotion^72^. This biological role for TLR4 in melanoma is partially in contrast with our observation of a better survival in melanoma patients with higher TLR4 levels.

Finally, we extended our molecular investigation to miRNA isoforms that were most abundant in our samples. According to the recent observation that miRNA isoforms can discriminate human cancers^36^, we detected a relevant number of miRNA variants in our dataset of single and multiple melanomas. We expected the canonical miRNAs (e.g., the sequences cataloged in miRBase), to be the most abundant isoform of each miRNA. However, we found and showed that some isomiRs were up to ten times more expressed than the canonical miRNA. We identified highly expressed isomiRs belonging to three important miRNA families: miR-30 family, miR-200 family, and miR-10 family. Canonical miRNAs, from these family, were thoroughly described as key regulators in cancer and melanoma^73–76^. The functional role of the isomiRs from these families is still unknown. We can speculate that, similar to protein-coding gene isoforms, which were discovered to play an important role in biological diversity and evolution^77^, isomiRs could have relevant functional roles as well. Cloonan et al. studied the role of two isomiRs of miR-10 family^23^, which were detected also in our dataset but with a low isomiR/canonical ratio: hsa-miR-10a-5p|+1|+1 (ratio = 0.12) and hsa-miR-10b-5p|+1|+1 (ratio = 0.26) (Supplementary Fig. 4). They suggested that the canonical miRNA works together with its isomiRs to increase the signal-to-noise ratio in miRNA–mRNA targeting^23^.

We analyzed two isomiRs (hsa-miR-125a-5p|0|−2 and hsa-miR-200b-3p|0|+1) highly expressed in our dataset. Specifically, we focused our analysis on the specific isoform of miR-125a-5p, lacking two nucleotides at the 3′-end, because it has a different expression trend in our groups (BN, CM, and MPM) if compared to its canonical form and resulted to be significantly differentially expressed in MPMs. This isoform is highly abundant in melanoma, as we confirmed by analyzing its levels across 32 tumor types from the TCGA database, and the ratio between miR-125a-5p isoform and the canonical form is broader in TCGA SKCM tumors (range 0.1–1100 times) and two to six logs more abundant in nevi and melanomas in our study. Moreover, we detected a specific dysregulation of the isoform, but not the canonical form, in multiple melanomas. Bioinformatic analyses revealed that miR-125a-5p shorted isoform putatively loses the ability to target and regulate a group of genes specifically involved in cell adhesion and cell differentiation. In particular, there seems to be lack of regulation of genes involved in neuronal differentiation. Indeed, miR-125a is the human ortholog of lin-4, the very first miRNA identified in *Caenorhabditis elegans* in 1993^78^. In mammals, miR-125 is expressed in embryonic stem cells and promote cell differentiation. Specifically, miR-125 has a specific role in adult nervous system development and neuronal differentiation^79,80^. We speculated that the imbalance between major miR-125 isoforms in melanocytes could reflect a major role for miR-125 in melanocyte development and differentiation from the neural crest^81^, differentiating this lineage from other common ancestor cells. This role could be consequently reflected in melanoma development and progression.

Overall, we provide here a comprehensive characterization of miRNA/isomiR dysregulation and regulatory network in single primary melanomas and MPMs. The pattern of miRNA alterations supports a less aggressive phenotype of MPMs, whereas isomiR-125a-5p levels proved to be enriched in melanoma and differentially expressed in MPMs, thus confirming the relevance of small noncoding RNA alterations in this fascinating—but poorly studied—melanoma subtype. Our observations about non-random dysregulation of specific miRNA isoforms in melanoma pose the basis for further functional studies.

## Supplementary information

Supplementary Figure legends

Supplementary Figure 1

Supplementary Figure 2

Supplementary Figure 3

Supplementary Figure 4

Supplementary Figure 5

Supplementary Table 1

Supplementary Table 2

Supplementary Table 3

Supplementary Table 4

Supplementary Table 5

Supplementary Table 6

## Data Availability

NGS raw data (FASTQ format) are available through European Nucleotide Archive (ENA) with the following accession number: PRJEB35819. The isomiR dataset used and analyzed in the current study is available from the corresponding author on reasonable request.
