# Supplementary material for: Unraveling the role of microRNA/isomiR network in multiple primary melanoma pathogenesis
Source: Cell Death Dis. 2021 May 12;12(5):473. doi: 10.1038/s41419-021-03764-y (PMC8115306; doi:10.1038/s41419-021-03764-y)
Supplement: Supplementary file 1 — Supplementary Figure legends [file 41419_2021_3764_MOESM1_ESM.pdf]

Unraveling the role of microRNA/isomiR networks in multiple primary melanoma pathogenesis

Emi Dika[1,2]*, Elisabetta Broseghini[1]*, Elisa Porcellini[1]*, Martina Lambertini[1,2], Mattia Riefolo[1], Giorgio Durante[1], Phillipe Loher[3], Roberta Roncarati[4,5], Cristian Bassi[4], Cosimo Misciali[2], Massimo Negrini[4], Isidore Rigoutsos[3], Eric Londin[3], Annalisa Patrizi[1,2], Manuela Ferracin[1]

1 Department of Experimental, Diagnostic and Specialty Medicine (DIMES), University of Bologna, Bologna, Italy.
2 Dermatology Unit, Sant'Orsola-Malpighi Hospital, Bologna, Italy.
3 Computational Medicine Center, Sidney Kimmel Medical College, Thomas Jefferson University, Philadelphia, PA, 19107, USA.
4 Department of Morphology, Surgery and Experimental Medicine, University of Ferrara, Ferrara, Italy.
5 CNR, Institute of Genetics and Biomedical Research, National Research Council of Italy, Milan, Italy.
Correspondence to manuela.ferracin@unibo.it
*These authors contributed equally to this work

This file includes：

Legends of Figure Supplementary 1 to Figure Supplementary 5

Legends of Supplementary Figures

Supplementary Figure 1. Metacore miRNA/targets network analysis of 22 miRNAs differently expressed in multiple primary melanoma (MPM) compared to cutaneous melanoma (CM). The maps obtained using MetaCore network analysis illustrate the genes involved in MPM specific biology. microRNA downregulated in MPM vs. CM are shown as blue circles, upregulated as red circles. Differentially expressed miRNAs result in the dysregulation of three target hubs: TLR4, ITGA6 and BTG2, whose expression is associated with a better prognosis in TCGA SKCM cohort (n=458).

Supplementary Figure 2. The -2nt shorter isoform of miR-125a-5p is more represented than the conventional form in our NGS data from benign nevi (BN), cutaneous melanoma (CM) and multiple primary melanoma (MPM). a. Sequence and chromosomal position of conventional and -2nt shorter isoform of miR-125a-5p. IGV representation of miR-125a-5p sequencing reads in exemplary BN, CM MPM samples shows that the reads for the conventional miR-125a-5p (24nt) are few or absent in comparison to the shorter isoforms (22-23nt). **b.** Differences between miRCURY LNA and miSCRIPT assay designs (Qiagen) allowed the quantification of the miR-125a-5p canonical form (24nt) and 5' isoforms or the quantification of all miR-125a-5p isoforms, respectively. The presence of a miRNA-specific reverse primer in miRCURY LNA assay allows to amplify and quantify only the canonical form of the miRNA, while the presence of a universal reverse primer in miSCRIPT Assay allows to amplify and quantify all miRNA isoforms, with different sequences at the 3' end.

Supplementary Figure 3. Representation of miR-30 family isoforms in all samples from small RNA seq data. a. miR-30 family contains 5 members and 6 mature microRNA molecules encoded by 6 genes distributed on three different chromosomes: miR-30e and miR-30c-1 (chr1), miR-30a and miR-30c-2 (chr6), miR-30b and miR-30d (chr8). Tables report the canonical microRNAs and all detected isomiRs, their sequences and the expression ratio between each isomiR and the canonical miRNA (if the sequence was detected). #: the canonical miRNA is not detected in our small RNA seq data. **b.** Expression ratio of each isomiR/miRNA is illustrated with bar chart. The most expressed isoform is a longer 3' isomiR for miR-30c-1, miR-30a and miR-30d. miR-30b isomiR is less expressed if compared to the canonical miRNA (ratio < 1). miR-30c-2 and miR-30e canonical miRNAs were not detected in these samples. miR-30e isomiRs can be considered "orphan isomiRs" in this dataset.

Supplementary Figure 4. Representation of miR-10 family isoforms in all samples from small RNA seq data. a miR-10 family consists of miR-10a and miR-10b (encoded in *Hox* clusters, chr17 and chr2 respectively), and the more distantly related miR-99a/b, miR-100 and miR-125 (chr21, chr11 and chr19). Tables report the canonical microRNAs and all detected isomiRs, their sequences and the expression ratio between each isomiR and the canonical miRNA (if the sequence was detected). #: the canonical miRNA is not detected in our small RNA seq data **b.** Expression ratio of each isomiR/miRNA is illustrated with bar chart. The most expressed isoform is a shorter 3' isomiR for miR-10a, miR-10a, miR-99a, miR-100, miR-125a. miR-125b and miR-99b isomiRs are less expressed if compared to the canonical miRNA (ratio < 1). miR-125b-2 canonical miRNA was not detected in these samples, and its isomiRs can be considered "orphan isomiRs" in this dataset.

Supplementary Figure 5. Representation of miR-200 family isoforms in all samples from small RNA seq data. a. miR-200 family contains 5 mature microRNA molecules encoded by 5 genes distributed on two different chromosomes: miR-200b, miR-200a and miR-429 (chr1), miR-200c and miR-141 (chr12). Tables report the canonical microRNAs and all detected isomiRs, their sequences and the expression ratio between each isomiR and the canonical miRNA (if the sequence was detected). #: the canonical miRNA is not detected in our small RNA seq data **b.** Expression ratio of each isomiR/miRNA is illustrated with bar chart. The most expressed isoform is a longer 3'isomiR for miR-200b and miR200a, while a shorter 3'isomiR for miR-141. miR-200c isomiR is less expressed if compared to the canonical miRNA (ratio < 1). miR-429 canonical miRNA was not detected in these samples, and its isomiR can be considered "orphan isomiRs" in this dataset.
