# Supplementary figures and images for: Unraveling the role of microRNA/isomiR network in multiple primary melanoma pathogenesis
Source: Cell Death Dis. 2021 May 12;12(5):473. doi: 10.1038/s41419-021-03764-y (PMC8115306; doi:10.1038/s41419-021-03764-y)

**a**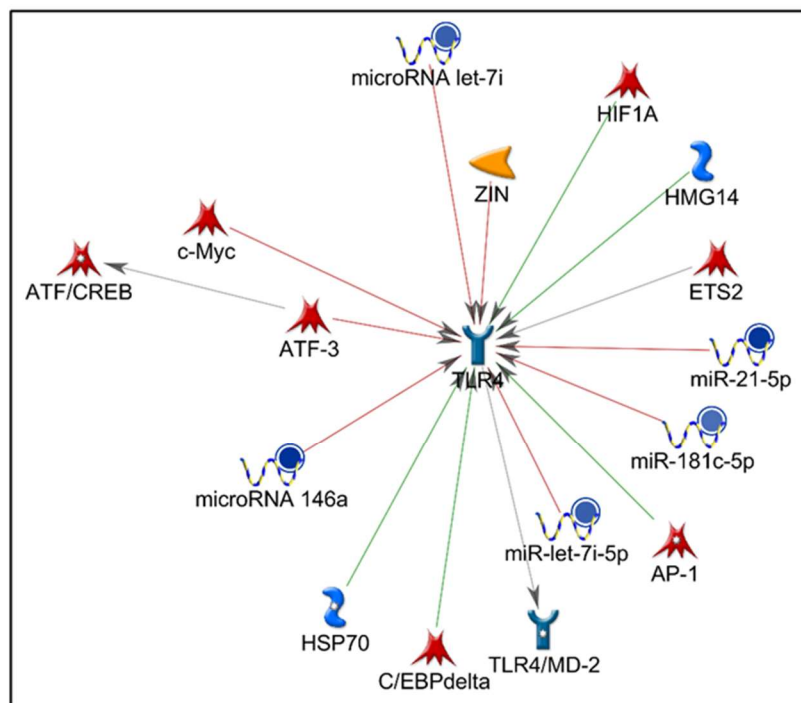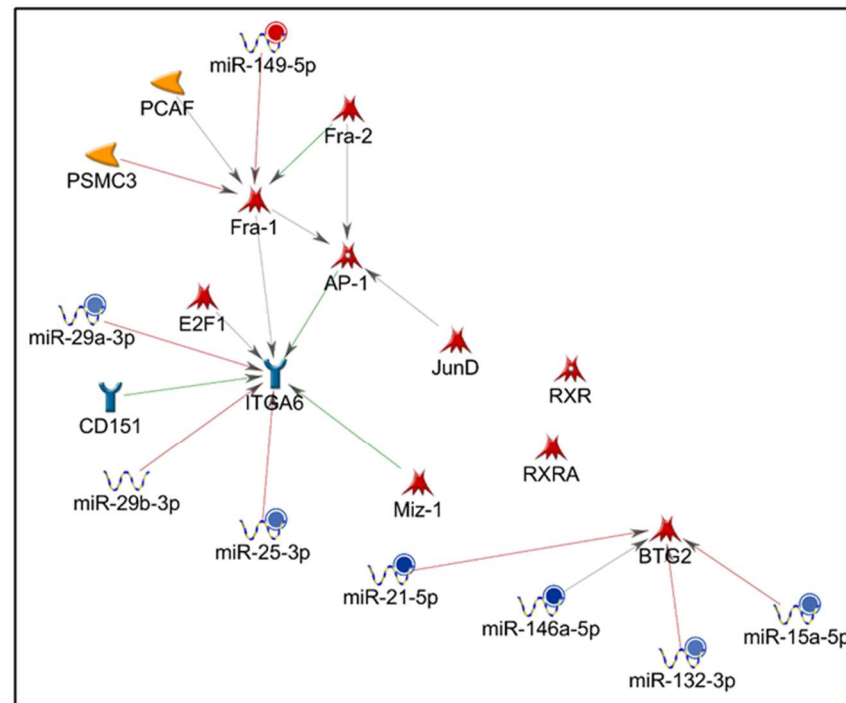**b**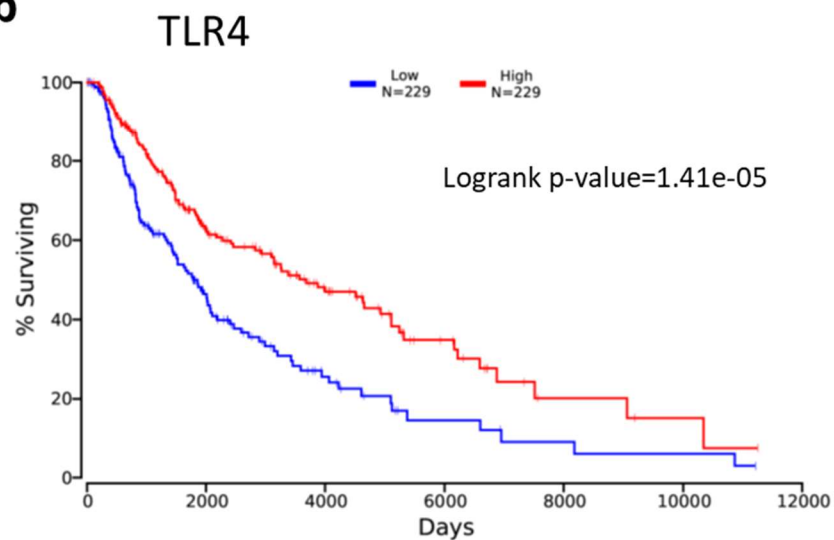**ITGA6**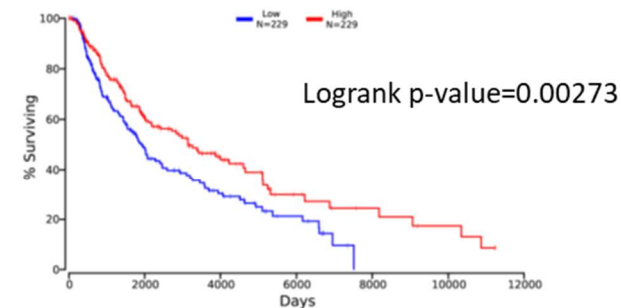**BTG2**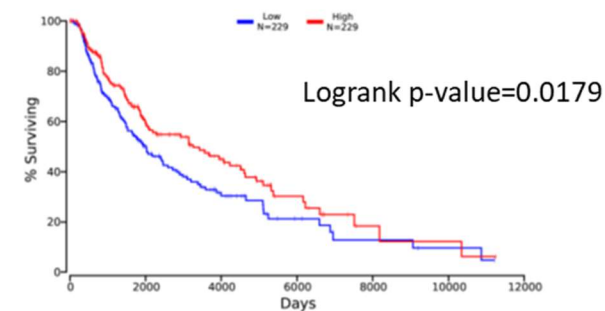

Supplement: Supplementary file 2 — Supplementary Figure 1 [file 41419_2021_3764_MOESM2_ESM.pdf]

# miR-10 family

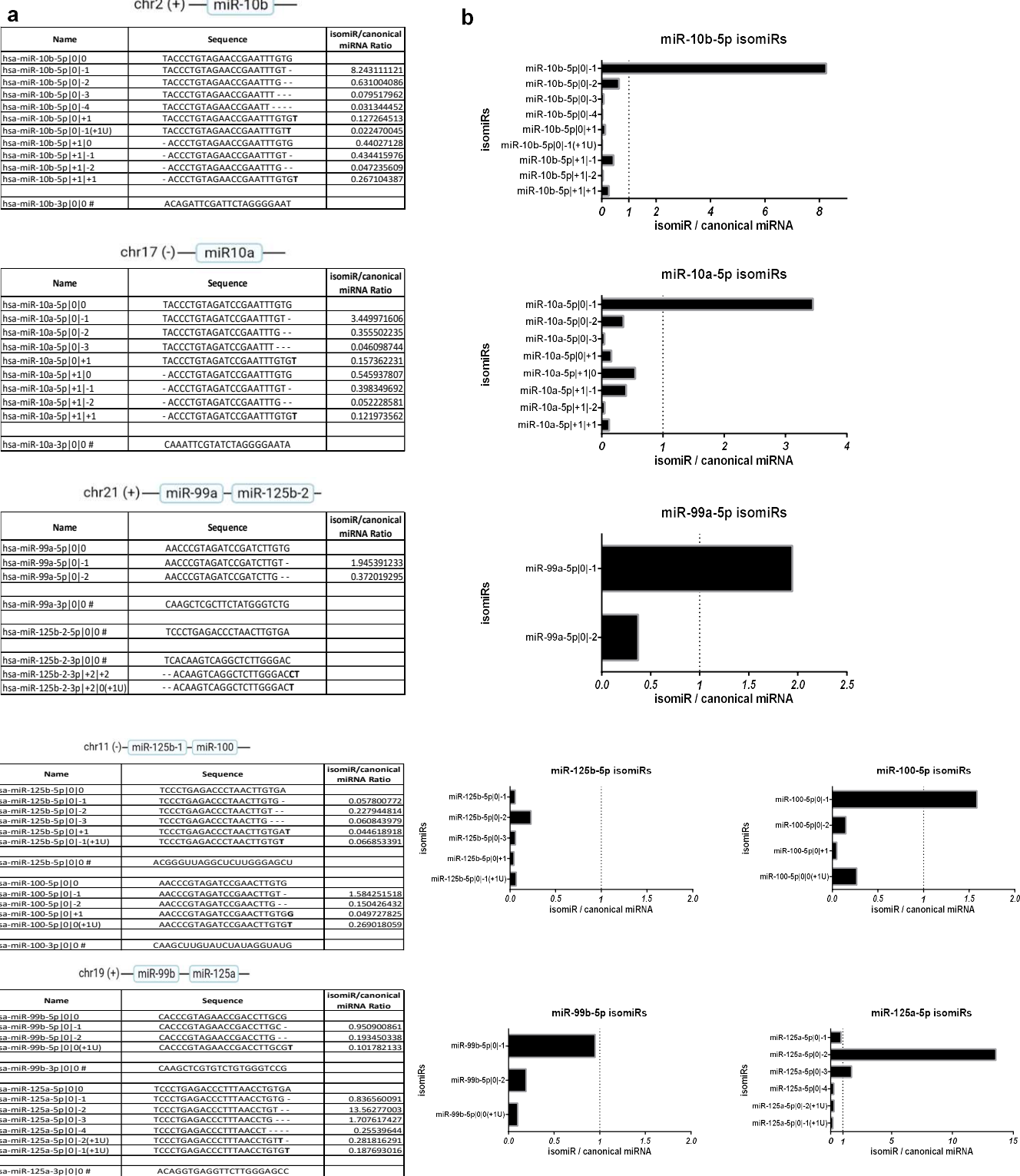

Supplement: Supplementary file 5 — Supplementary Figure 4 [file 41419_2021_3764_MOESM5_ESM.pdf]
