# Supplementary material for: Unraveling the role of microRNA/isomiR network in multiple primary melanoma pathogenesis
Source: Cell Death Dis. 2021 May 12;12(5):473. doi: 10.1038/s41419-021-03764-y (PMC8115306; doi:10.1038/s41419-021-03764-y)
Supplement: Supplementary file 3 — Supplementary Figure 2 [file 41419_2021_3764_MOESM3_ESM.pdf]

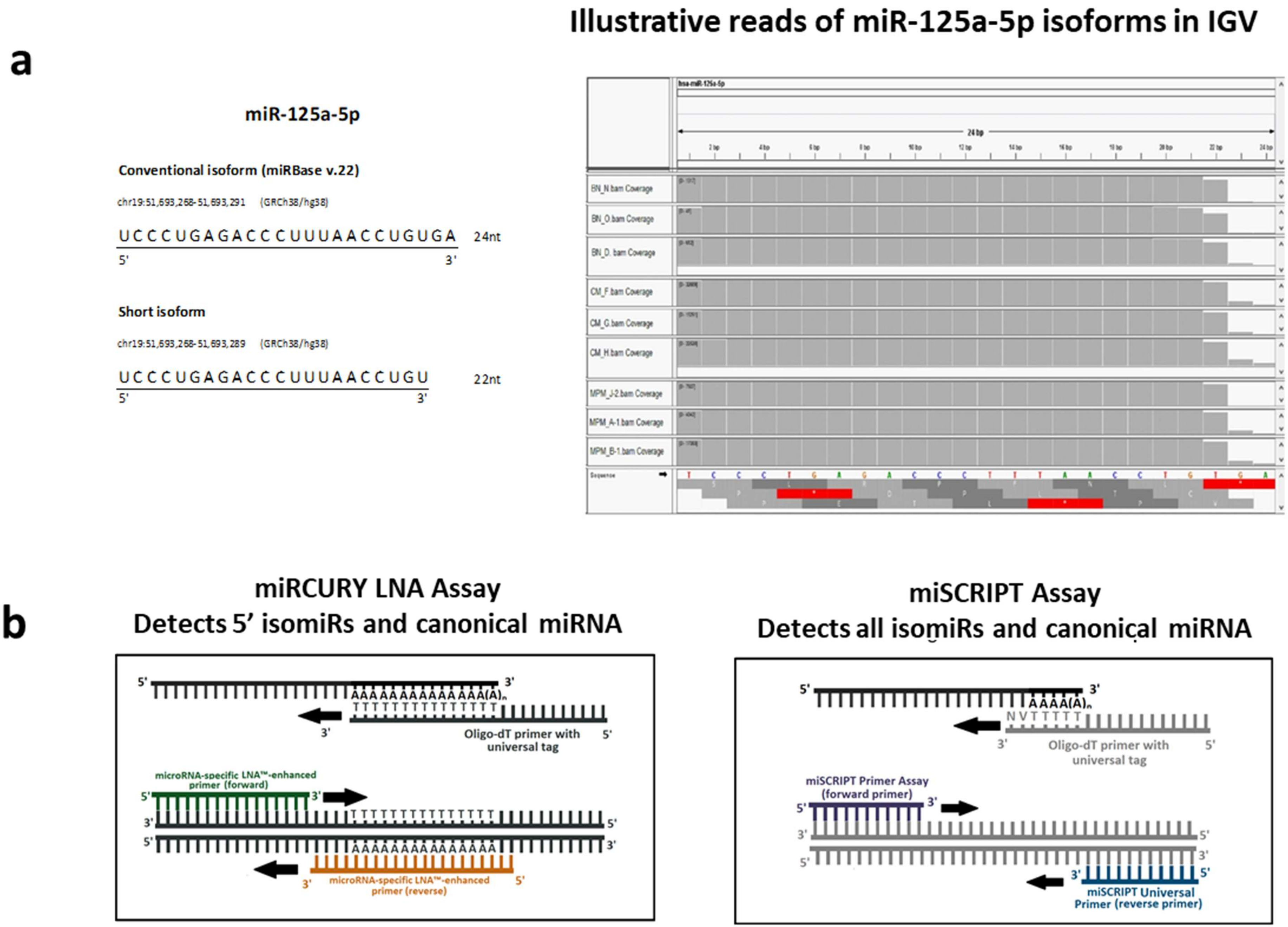

Illustrative reads of miR-125a-5p isoforms in IGV
a
miR-125a-5p
Conventional isoform (miRBase v.22)
chr19:51,693,268-51,693,291 (GRCh38/hg38)
UCCCUGAGACCCUUUAACCUGUGA 24nt
5' 3'
Short isoform
chr19:51,693,268-51,693,289 (GRCh38/hg38)
UCCCUGAGACCCUUUAACCUGU 22nt
5' 3'
b
miRCURY LNA Assay
Detects 5' isomiRs and canonical miRNA
AAAAAAAAAAAAAA(A)n
Oligo-dT primer with universal tag
microRNA-specific LNA™-enhanced primer (forward)
microRNA-specific LNA™-enhanced primer (reverse)
miSCRIPT Assay
Detects all isomiRs and canonical miRNA
AAAA(A)n
NVTTTTT
Oligo-dT primer with universal tag
miSCRIPT Primer Assay (forward primer)
miSCRIPT Universal Primer (reverse primer)
