# Supplementary material for: Unraveling the role of microRNA/isomiR network in multiple primary melanoma pathogenesis
Source: Cell Death Dis. 2021 May 12;12(5):473. doi: 10.1038/s41419-021-03764-y (PMC8115306; doi:10.1038/s41419-021-03764-y)

miR-30 family

a

chr1 (+) — miR-30e — miR-30c-1 —

Name	Sequence	isomiR/canonical miRNA Ratio
hsa-miR-30e-5p|0|0 #	TGTAAACATCCTTGACTGGAAG	
hsa-miR-30e-5p|0|-1	TGTAAACATCCTTGACTGGAA -	
hsa-miR-30e-5p|0|-2	TGTAAACATCCTTGACTGGA - -	
hsa-miR-30e-5p|0|+1	TGTAAACATCCTTGACTGGAAG**C**	
hsa-miR-30e-5p|0|+2	TGTAAACATCCTTGACTGGAAG**CT**	
hsa-miR-30e-5p|0|+2(+1U)	TGTAAACATCCTTGACTGGAAG**CTT**	
hsa-miR-30e-5p|+1|+2	- GTAAACATCCTTGACTGGAAG**CT**	
hsa-miR-30e-3p|0|0	CTTTCAGTCGGATGTTTACAGC	
hsa-miR-30c-5p|0|0	TGTAAACATCCTACACTCTCAGC	
hsa-miR-30c-5p|0|+1	TGTAAACATCCTACACTCTCAGC**T**	4.419753371

chr6 (-) — miR-30a — miR-30c-2 —

Name	Sequence	isomiR/canonical miRNA Ratio
hsa-miR-30a-5p|0|0	TGTAAACATCCTCGACTGGAAG	
hsa-miR-30a-5p|0|-1	TGTAAACATCCTCGACTGGAA -	2.11398921
hsa-miR-30a-5p|0|-2	TGTAAACATCCTCGACTGGA - -	5.411779755
hsa-miR-30a-5p|0|+1	TGTAAACATCCTCGACTGGAAG**C**	3.427772113
hsa-miR-30a-5p|0|+2	TGTAAACATCCTCGACTGGAAG**CT**	15.90352778
hsa-miR-30a-5p|0|0(+1U)	TGTAAACATCCTCGACTGGAAG**T**	0.788466371
hsa-miR-30a-5p|+1|+2	- GTAAACATCCTCGACTGGAAG**CT**	0.546994708
hsa-miR-30a-3p|0|0	CTTTCAGTCGGATGTTTGCAGC	
hsa-miR-30c-2-3p|0|0 #	CTGGGAGAAGGCTGTTTACTCT	

chr8 (-) — miR-30b — miR-30d —

Name	Sequence	isomiR/canonical miRNA Ratio
hsa-miR-30b-5p|0|0	TGTAAACATCCTACACTCAGCT	
hsa-miR-30b-5p|0|-1	TGTAAACATCCTACACTCAGC -	0.552310527
hsa-miR-30b-3p|0|0 #	CTGGGAGGTGGATGTTTACTTC	
hsa-miR-30d-5p|0|0	TGTAAACATCCCCGACTGGAAG	
hsa-miR-30d-5p|0|-1	TGTAAACATCCCCGACTGGAA -	2.00625
hsa-miR-30d-5p|0|-2	TGTAAACATCCCCGACTGGA - -	1.745275673
hsa-miR-30d-5p|0|+1	TGTAAACATCCCCGACTGGAAG**C**	3.38984638
hsa-miR-30d-5p|0|+2	TGTAAACATCCCCGACTGGAAG**CT**	10.08834175
hsa-miR-30d-5p|0|0(+1U)	TGTAAACATCCCCGACTGGAAG**T**	0.351083754
hsa-miR-30d-5p|0|+2(+1U)	TGTAAACATCCCCGACTGGAAG**CTT**	0.251441498
hsa-miR-30d-3p|0|0 #	CUUUCAGUCAGAUGUUUGCUGC	

b

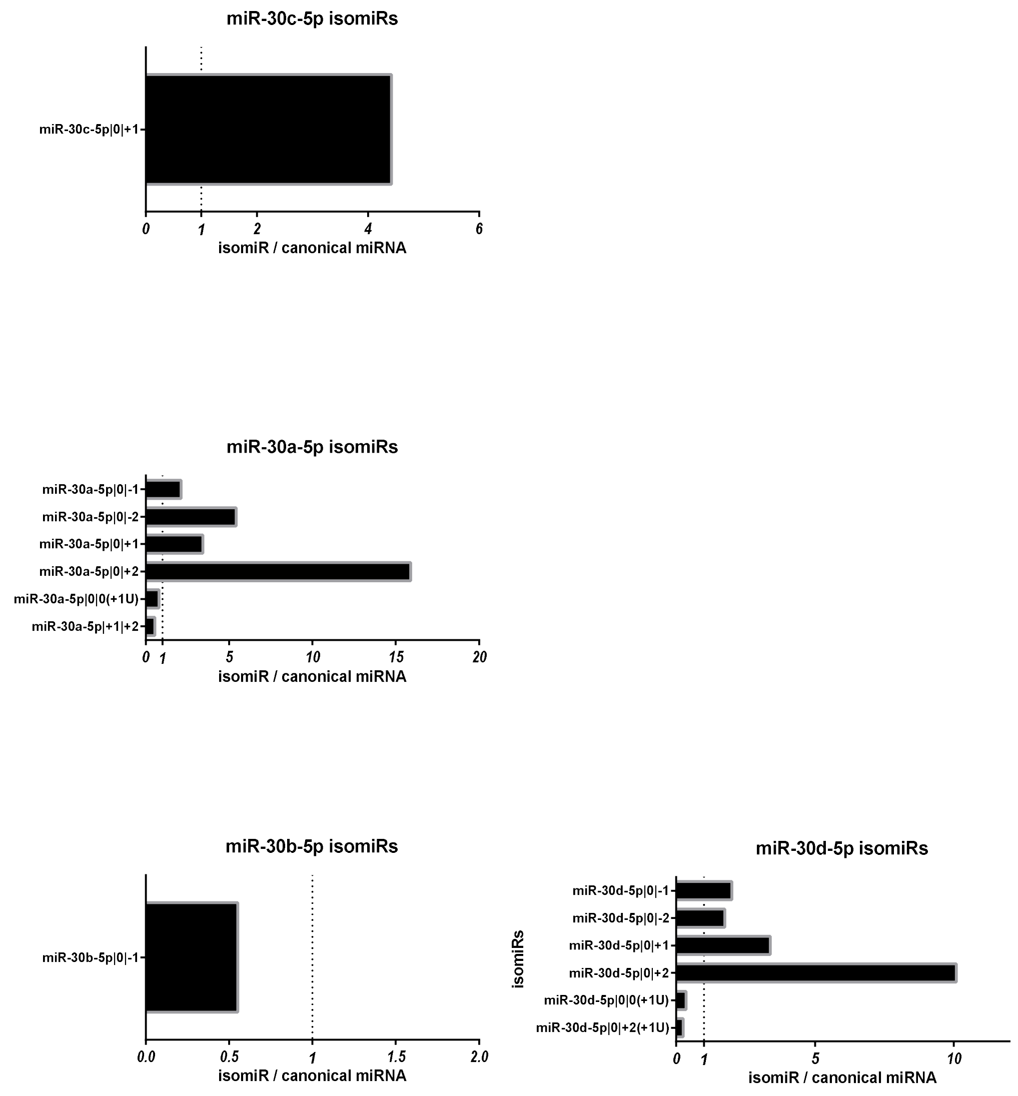

Supplement: Supplementary file 4 — Supplementary Figure 3 [file 41419_2021_3764_MOESM4_ESM.pdf]
