# Supplementary material for: Unraveling the role of microRNA/isomiR network in multiple primary melanoma pathogenesis
Source: Cell Death Dis. 2021 May 12;12(5):473. doi: 10.1038/s41419-021-03764-y (PMC8115306; doi:10.1038/s41419-021-03764-y)

miR-200 family

a

chr1 (+) — miR-200b — miR-200a — miR-429 —

Name	Sequence	isomiR/canonical miRNA Ratio
hsa-miR-200b-5p|0|0 #	CATCTTACTGGGCAGCATTGGA	
hsa-miR-200b-3p|0|0	TAATACTGCCTGGTAATGATGA	
hsa-miR-200b-3p|0|+1	TAATACTGCCTGGTAATGATGA**C**	3.384096919
hsa-miR-200b-3p|0|+1(+1U)	TAATACTGCCTGGTAATGATGA**CT**	2.493702431
hsa-miR-200b-3p|+1|+1(+1U)	- AATACTGCCTGGTAATGATGA**CT**	1.058009474
hsa-miR-200a-5p|0|0 #	CATCTTACCGGACAGTGCTGGA	
hsa-miR-200a-3p|0|0	TAACACTGTCTGGTAACGATGT	
hsa-miR-200a-3p|0|-1	TAACACTGTCTGGTAACGATG -	0.796067898
hsa-miR-200a-3p|0|+1	TAACACTGTCTGGTAACGATGT**T**	1.26819602
hsa-miR-429|0|0 #	TAATACTGTCTGGTAAAACCGT	
hsa-miR-429|0|-1	TAATACTGTCTGGTAAAACCG -	

chr12 (+) — miR-200c — miR-141 —

Name	Sequence	isomiR/canonical miRNA Ratio
hsa-miR-200c-5p|0|0 #	CGTCTTACCCAGCAGTGTTTGG	
hsa-miR-200c-3p|0|0	TAATACTGCCGGGTAATGATGGA	
hsa-miR-200c-3p|0|-1	TAATACTGCCGGGTAATGATGG -	0.516753753
hsa-miR-141-5p|0|0 #	CATCTTCCAGTACAGTGTTGGA	
hsa-miR-141-3p|0|0	TAACACTGTCTGGTAAAGATGG	
hsa-miR-141-3p|0|-1	TAACACTGTCTGGTAAAGATG -	23.15295859
hsa-miR-141-3p|0|+1	TAACACTGTCTGGTAAAGATGG**C**	1.946459168
hsa-miR-141-3p|0|+2	TAACACTGTCTGGTAAAGATGG**CT**	0.779214083

b

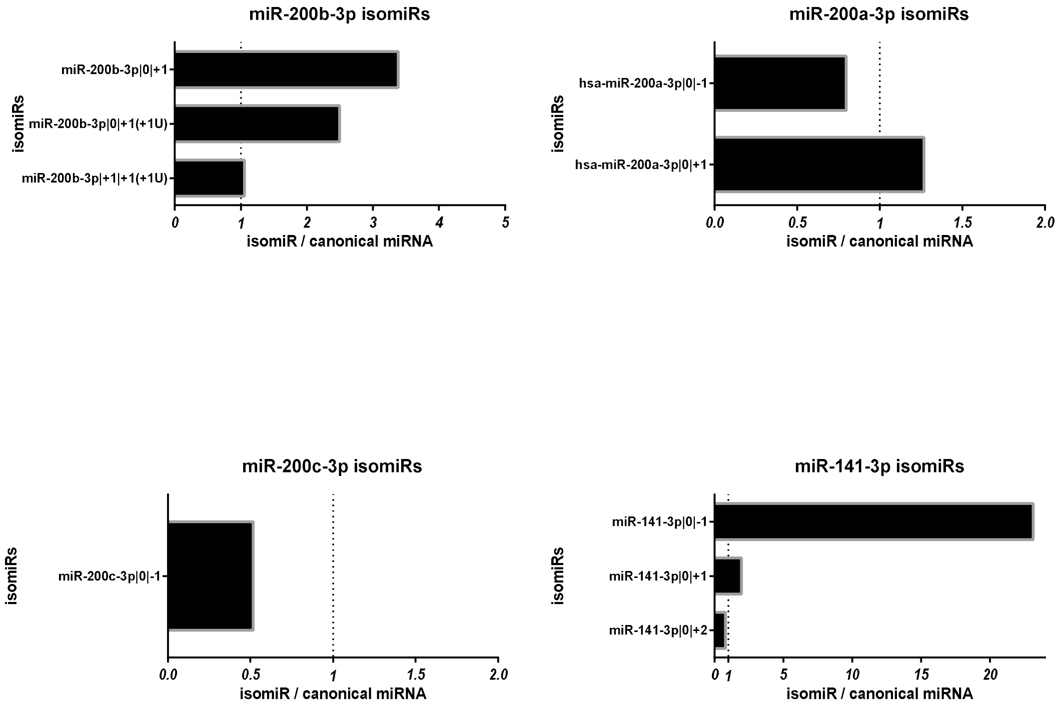

Supplement: Supplementary file 6 — Supplementary Figure 5 [file 41419_2021_3764_MOESM6_ESM.pdf]
