# Supplementary material for: Unraveling the role of microRNA/isomiR network in multiple primary melanoma pathogenesis
Source: Cell Death Dis. 2021 May 12;12(5):473. doi: 10.1038/s41419-021-03764-y (PMC8115306; doi:10.1038/s41419-021-03764-y)
Supplement: Supplementary file 7 — Supplementary Table 1 [file 41419_2021_3764_MOESM7_ESM.pdf]

Supplementary Table 1 – List of microRNAs differentially expressed in multiple vs. primary melanoma

microRNA	adjusted p-value	Regulation MPM vs. CM	Fold change
hsa-let-7i-5p	0.0153	down	-2.7
hsa-miR-106b-3p	0.0347	down	-2.3
hsa-miR-132-3p	0.045	down	-1.8
hsa-miR-146a-5p	0.025	down	-4.8
hsa-miR-15a-5p	0.0314	down	-2.3
hsa-miR-181a-3p	0.0153	down	-2.7
hsa-miR-181c-5p	0.036	down	-2
hsa-miR-21-3p	0.0153	down	-4.7
hsa-miR-21-5p	0.0205	down	-4.3
hsa-miR-25-3p	0.0084	down	-2.2
hsa-miR-29a-3p	0.0205	down	-1.7
hsa-miR-340-5p	0.0341	down	-2.4
hsa-miR-3614-5p	0.0135	down	-3.2
hsa-miR-532-5p	0.0341	down	-2.1
hsa-miR-584-5p	0.0167	down	-6.3
hsa-miR-651-5p	0.0275	down	-1.7
hsa-miR-941	0.0318	down	-1.8
hsa-miR-125b-2-3p	0.0314	up	2.1
hsa-miR-125b-5p	0.0398	up	2.4
hsa-miR-149-5p	0.0205	up	3.2
hsa-miR-205-5p	0.0344	up	2.1
hsa-miR-99a-5p	0.0314	up	2.4
