# Supplementary material for: Unraveling the role of microRNA/isomiR network in multiple primary melanoma pathogenesis
Source: Cell Death Dis. 2021 May 12;12(5):473. doi: 10.1038/s41419-021-03764-y (PMC8115306; doi:10.1038/s41419-021-03764-y)
Supplement: Supplementary file 8 — Supplementary Table 2 [file 41419_2021_3764_MOESM8_ESM.pdf]

Supplementary Table 2 - List of microRNAs differentially expressed in paired multiple melanomas from the same patient

microRNA	p-value (paired)	Regulation 1st MPM vs. 2nd MPM	Fold change
hsa-let-7d-3p	0.0174	down	-1.8
hsa-let-7e-5p	0.0153	down	-2
hsa-miR-1226-3p	0.0345	down	-3.1
hsa-miR-1249	0.0338	down	-1.8
hsa-miR-125a-5p	0.0063	down	-1.9
hsa-miR-1269b	0.0254	down	-2
hsa-miR-145-3p	0.0313	down	-1.7
hsa-miR-149-5p	0.0157	down	-1.7
hsa-miR-200b-3p	0.0134	down	-1.8
hsa-miR-224-5p	0.0135	down	-1.8
hsa-miR-320b	0.0243	down	-2
hsa-miR-328-3p	0.0103	down	-1.9
hsa-miR-330-5p	0.006	down	-1.5
hsa-miR-365a-5p	0.0398	down	-1.6
hsa-miR-375	0.0166	down	-1.8
hsa-miR-433-3p	0.0019	down	-2.2
hsa-miR-4423-3p	0.009	down	-2
hsa-miR-505-3p	0.0194	down	-1.8
hsa-miR-6511b-3p	0.0299	down	-2
hsa-miR-671-3p	0.0431	down	-1.6
hsa-miR-8058	0.0334	down	-2.2
hsa-miR-877-5p	0.016	down	-2.1
hsa-miR-92a-1-5p	0.0464	down	-1.6
hsa-miR-92b-3p	0.01	down	-1.6
hsa-miR-96-5p	0.0062	down	-2.6
hsa-miR-98-3p	0.0246	down	-1.8
hsa-miR-99b-5p	0.0054	down	-1.6
hsa-miR-1248	0.0423	up	3.4
hsa-miR-223-3p	0.0458	up	1.8
hsa-miR-2392	0.0362	up	2.3
hsa-miR-3607-5p	0.0308	up	3.1
hsa-miR-3609	0.0028	up	2.5
hsa-miR-4286	0.0071	up	1.9
hsa-miR-4466	0.019	up	2
hsa-miR-7641	0.0129	up	2.1
hsa-miR-887-3p	0.0441	up	2
hsa-miR-99a-3p	0.0342	up	1.6
