# Supplementary material for: Unraveling the role of microRNA/isomiR network in multiple primary melanoma pathogenesis
Source: Cell Death Dis. 2021 May 12;12(5):473. doi: 10.1038/s41419-021-03764-y (PMC8115306; doi:10.1038/s41419-021-03764-y)
Supplement: Supplementary file 9 — Supplementary Table 3 [file 41419_2021_3764_MOESM9_ESM.pdf]

Supplementary Table 3 - Pathway enrichment analysis of 22 microRNAs differentially expressed in CM vs. MPM

	**Enrichment by Pathway Maps**					
**#**	**Maps**	**p-value**	**FDR**	**Total**	**In Data**	**Network Objects from Active Data**
1	microRNA in Prostate Cancer	3.1729E-10	8.884E-09	51	6	microRNA 125b, microRNA 15a, microRNA 205, microRNA 106b, microRNA 21, microRNA 146a
2	Development_MicroRNA-dependent regulation of EMT	1.0715E-05	0.000144	24	3	miR-205-5p, miR-205-3p, microRNA 205
3	Brca1 in ovarian cancer	1.5429E-05	0.000144	27	3	miR-146a-3p, microRNA 146a, miR-146a-5p
4	NRSF-dependent transcription deregulation in Huntington's Disease	4.7521E-05	0.0003326	39	3	microRNA 29a, microRNA 132, miR-132-3p
5	Suppression of p53 signaling in multiple myeloma	0.00010059	0.0004694	50	3	miR-181a-5p, miR-25-3p, miR-106b-5p
6	TGF-beta signaling via microRNA in breast cancer	0.00010059	0.0004694	50	3	miR-181a-5p, microRNA 21, miR-21-5p
7	PR action in breast cancer: stimulation of metastasis	0.00060342	0.0024137	20	2	microRNA 29a, miR-29a-3p
8	Signal transduction_Angiotensin II/ AGTR1 signaling via TGF-beta 1 and SMADs	0.0026682	0.0093387	42	2	microRNA 21, miR-21-5p
9	Anti-apoptotic action of ErbB2 in breast cancer	0.00391176	0.0121699	51	2	miR-15a-5p, microRNA 15a
10	MicroRNAs in melanoma	0.00486402	0.0128091	57	2	miR-205-5p, miR-532-5p
11	Hyaluronic acid/ CD44 signaling in cancer	0.00503213	0.0128091	58	2	microRNA 21, miR-21-5p
12	Regulation of microRNAs in colorectal cancer	0.00573111	0.0133726	62	2	microRNA 21, miR-21-5p
13	ErbB2-induced breast cancer cell invasion	0.00666379	0.0143528	67	2	microRNA 21, miR-21-5p
14	Hypertrophy of asthmatic airway smooth muscle cells	0.0072544	0.0145088	70	2	miR-25-3p, microRNA 25
15	Stem cells_Hypothetical role of microRNAs in fibrosis development after myocardial infarction	0.04686304	0.0874777	26	1	miR-21-5p
16	Upregulation of MITF in melanoma	0.06432291	0.1125651	36	1	miR-340-5p
17	Development_Regulation of lung epithelial progenitor cell differentiation	0.07293794	0.1140681	41	1	miR-106b-5p
18	Transcription targets of Androgen receptor involved in Prostate Cancer	0.07465184	0.1140681	42	1	microRNA 125b
19	Signal transduction_Angiotensin II/ AGTR1 signaling via JAK/STAT	0.0797754	0.1140681	45	1	microRNA 21
20	K-RAS signaling in lung cancer	0.08147723	0.1140681	46	1	microRNA 21
